# Functional roles of magnetic iron oxide nanoparticles in mixed matrix membrane technology for wastewater treatment: a critical review

**DOI:** 10.1039/d6ra00618c

**Published:** 2026-04-07

**Authors:** Lamyaa A. Abdulridha, Khalid T. Rashid, Mohammed Ahmed Shehab, Najah Saeed Abdulridha, Adnan A. AbdulRazak

**Affiliations:** a Membrane Technology Research Unit, College of Chemical Engineering, University of Technology-Iraq Al Sinaa Street 52 10066 Baghdad Iraq che.23.05@grad.uotechnology.edu.iq Khalid.T.Rashid@uotechnology.edu.iq; b Gas Processes and Petrochemicals Engineering Department, Basrah University for Oil and Gas 61004 Basrah Iraq mohammed.ahmed@buog.edu.iq; c Oil and Gas Engineering Department, Basrah University for Oil and Gas 61004 Basrah Iraq n.saeed@buog.edu.iq; d Department of Oil and Gas Refining Engineering, Collage of Engineering, Al-Turath University Baghdad Iraq adnansss2002@yahoo.com

## Abstract

Magnetic nanoparticles (MNPs) have emerged as highly promising functional additives in mixed-matrix membranes (MMMs) for advanced wastewater treatment. Their unique magnetic responsiveness, large surface area, tunable surface chemistry, and catalytic activity enable enhanced pollutant removal efficiency, membrane permeability, and antifouling performance. This review highlights the fundamental properties of common MNPs such as magnetite (Fe_3_O_4_) and maghemite (γ-Fe_2_O_3_). It discusses their incorporation into various polymeric membranes to target heavy metals, organic dyes, pharmaceuticals, and pathogens. The mechanisms governing pollutant separation, including adsorption, catalytic degradation, and magnetic-assisted separation, are examined in detail. Additionally, the effects of particle loading, surface modification, and dispersion techniques on membrane structural integrity and filtration performance are analyzed. Challenges such as nanoparticle leaching, stability under operational conditions, scaling-up synthesis, and environmental safety considerations are also evaluated. Finally, MNP-embedded MMMs offer a promising pathway toward efficient, selective, and energy-saving wastewater purification technologies.

## Abstract 2

Roles of magnetic mixed matrix membrane for wastewater treatment.

## Graphical abstract

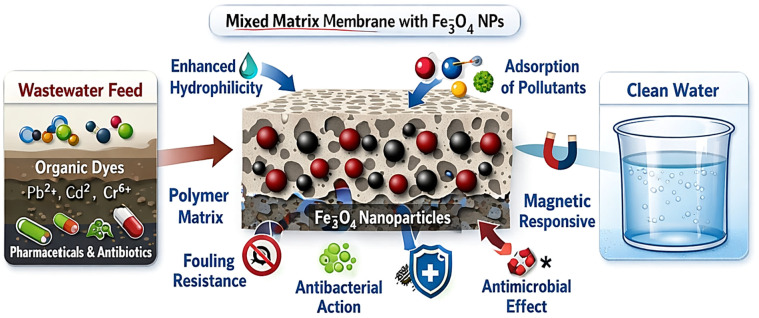

## 1 Introduction

Rapid advancements in wastewater treatment technologies have been fuelled by growing strain on freshwater resources and increasingly stringent discharge rules. Because of their high removal efficiency, small footprint, and scalability, membrane processes, especially polymeric ultrafiltration and nanofiltration remain problematic due to fouling, inadequate selectivity for some pollutants, and trade-offs between rejection and permeability.^1^ Many of these restrictions may be addressed by mixed matrix membranes (MMMs), which combine the processability of polymers with the functionality of nanoparticles.^2^ Particularly useful additions for MMMs are magnetic iron-oxide nanoparticles, mainly magnetite (Fe_3_O_4_) and maghemite (γ-Fe_3_O_4_). Their variable surface chemistry enables adsorption, catalytic/photocatalytic degradation, and increased hydrophilicity when appropriately surface-functionalized, while their innate magnetic responsiveness facilitates easy recovery and positioning under external fields. Fe-oxide nanoparticles have been extensively studied for dye adsorption, heavy-metal capture, antifouling modification, and photocatalytic pollutant destruction in water treatment applications due to their multifunctional properties.^3^ Fe_3_O_4_-based nanofillers have shown quantifiable improvements in water flux, fouling resistance, and contaminant removal when embedded in membrane matrices (such as PES, PSf, or PVDF), provided nanoparticle dispersion, polymer-particle compatibility, and particle immobilization (to prevent leaching) are carefully controlled. Careful surface modification (PVP, silica, polymers, or graphene oxide hybrids) stabilizes nanoparticles in the casting dope and promotes desired membrane morphologies and surface properties, as demonstrated by several experimental studies.^4^ Despite these benefits, significant obstacles remain before magnetic-nanoparticle-based MMMs can be developed into reliable, long-lasting treatment systems. These obstacles include nanoparticle agglomeration during membrane fabrication, possible nanoparticle leaching during operation, effects on mechanical integrity, and scaling up repeatable surface functionalization routes. Additionally, magnetic membrane bioreactors and magnetic-field-assisted operation offer promising avenues for process intensification; however, before industrial implementation, a thorough techno-economic and environmental study is necessary.^5^

This review methodically covers Fe-oxide nanoparticle synthesis and surface-functionalization techniques, their integration strategies into polymeric membranes, their mechanistic roles in fouling mitigation and pollutant removal, and the remaining obstacles to safe deployment and scale-up. Numerous review articles have been published on the use of magnetic materials as adsorbents and in biomedical applications.^6,7^ However, to the best of the authors' knowledge, there are no review papers specifically addressing the incorporation of these materials into membranes. This review establishes a focused research roadmap to accelerate the translation of magnetic nanoparticle-enabled mixed matrix membranes (MMMs) from laboratory studies to practical wastewater treatment applications. It critically addresses key barriers to deployment, including insufficient understanding of long-term operational stability, nanoparticle leaching and environmental risks, and the lack of integration with scalable membrane fabrication and process design. Emphasis is placed on polymer membranes incorporating charged additives, particularly iron oxide nanoparticles, with a detailed analysis of how their surface charge, particle size, and dispersion govern membrane morphology, separation efficiency, fouling resistance, and interfacial compatibility. The multifunctional benefits of iron oxides such as enhanced permeability, selectivity, and stimuli responsiveness are systematically evaluated alongside inherent challenges including aggregation and durability under realistic operating conditions. Comparative assessment with other charged fillers further underscores the unique advantages of iron oxides. Finally, critical knowledge gaps and future research priorities are identified to support the rational design and sustainable deployment of next generation charged MMMs.

## 2 Overview of magnetic iron oxide nanoparticles (Fe_3_O_4_, γ-Fe_2_O_3_, and **α**-Fe_2_O_3_)

Nanomaterials can be defined as materials that have one dimension at least ≤100 nm. It can be used in various applications based on its characteristics, especially high surface area-to-volume ratio. However, the aggregation problem that exists in nanoparticle media is usually overcome by functionalization, which modifies the surface chemistry of nanoparticles. There are many shapes and structures of nanomaterials, ranging from zero dimensions (0-D), such as quantum dots and nanoparticles, to one dimension (1-D), such as nanowires and nanopillars, to two dimensions (2-D), such as nanosheets and nanopores, and to three dimensions (3-D), such as complex hierarchical structures.^8,9^ Nowadays, a branch of hybrid nanoparticles, called core–shell nanoparticles, has given rise to astonishing physicochemical, optical, and biological properties. They comprise one or more shell layers and a core.^10^ The overall properties are determined by the core, which is chemically distinct from the other parts of the nanoparticle. The synthesis of these materials follows two strategies: top-down and bottom-up, where in the top-down strategy, the bulk material is converted to a nano-sized particle, while self-assembly of atoms governs the bottom-up approach to form the nano-sized particle (preferred for core–shell nanoparticles).^11,12^

**Fig. 1:**
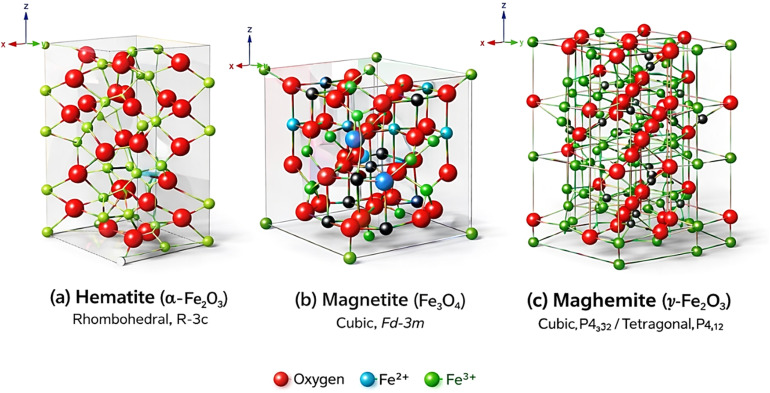
Crystal structure of iron oxides reproduced from ref. 13 with permission from University of Technology-Iraq, Copyright © 2023. Modifications were made using Gemini AI-assisted tool.

In recent years, magnetic nanoparticles have been widely investigated due to their superior electrical, magnetic, mechanical, and optical properties. Magnetic iron oxide nanoparticles are essential and the main materials in this field. These materials can be found in nature in many forms, but the most relevant are magnetite (Fe_3_O_4_), maghemite (γ-Fe_2_O_3_), and hematite (α-Fe_2_O_3_) as shown in (Fig. 1).^13^

The magnetite is known in nature as the black iron oxide. It has a face-centered cubic structure with an inverse spinel arrangement, containing both divalent and trivalent iron. It shows the strongest magnetic power among other transition-metal oxides.^14^ While maghemite is found in soils, it has a cubic spinel structure. The O^2−^ ions that are found in the unit maghemite crystal structure produce a cubic shape arrangement, while Fe^3+^ ions are distributed over tetrahedral and octahedral sites.^15^ Hematite is very common in soil and rocks and has several names, such as ferric oxide and red ochre. Actually, two-thirds of the octahedral sites are filled and occupied by Fe^3+^ ions. It is used in catalysts, gas sensors, and as a base material in preparing (Fe_3_O_4_) and (γ-Fe_2_O_3_).^16^

It is worth mentioning that magnetic iron oxide nanoparticles are strongly affected by their size, shape, and structure, and all these characteristics are controlled by the synthesis method, which determines their function and where they are applied. In general, the physical properties of the three types of iron oxide are shown in Table 1.^17^

**Table 1:** Iron oxides' physical characteristics reproduced from ref. 17 with permission from IntechOpen, Copyright © 2018

Properties	Magnetite	Maghemite	Hematite
Formula	Fe_3_O_4_	γ-Fe_2_O_3_	α-Fe_2_O_3_
Density (g cm^−3^)	5.18	4.87	5.26
Melting point (°C)	1583–1597	—	1350
Hardness	5.5	5	6.5
Type of magnetism	Ferrimagnetic	Ferrimagnetic	Antiferromagnetic or weak ferromagnetic
Curie temperature (K)	850	820–986	956
Saturation magnetization (Ms) at 300 K (A m^−2^ kg^−1^)	91–100	60–80	0.3
Standard free energy of formation 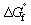 (KJ mol^−1^)	−1012.6	−711.1	−742.7
Crystallographic system	Cubic	Tetragonal	Rhombohedral, hexagonal
Structure	Inverse spinel	Defect spinel	Corundum
Space group	*Fd*3*m*	*P*4_3_32 (cubic)	*R*3*c* (hexagonal)
		*P*4_1_2_1_2 (tetragonal)	
Lattice parameter (nm)	*a* = 0.8396	*a* = 0.83474 (cubic)	*a* = 0.5034, *c* = 1.375 (hexagonal), *c* = 2.501 (tetragonal) *a*_Rh_ = 0.5427, *α* = 55.3° (rhombohedral)
		*a* = 0.8347, *c* = 2.501 (tetragonal)	

## 3 Synthesis methods of magnetic iron oxide NPs

### 3.1 Classic synthesis methods

A wide range of synthesis routes have been extensively explored and applied across diverse fields as illustrated in (Table 2), including co-precipitation,^18,19^ thermal decomposition,^20^ hydrothermal synthesis,^21^ electrochemical deposition,^22^ microemulsion techniques,^23^ laser pyrolysis,^24^ sonochemical methods,^25^ radiolytic reduction,^26^ microwave-assisted synthesis,^27^ flame spray pyrolysis,^28^ and chemical vapor deposition.^29^

**Table 2:** Performance of some magnetic iron oxide nanoparticles in different applications

Magnetic nanoparticles	Synthesis method	Application	Performance	Ref.
Iron oxide	Co-precipitation	Adsorption of methylene blue (MB)	Entrapment efficiency about 87% with approximately 3.638% (highest loading capacity) of MB	18
Fe_3_O_4_	Co-precipitation	Adsorption of Au(iii), Pd(ii), and Pt(iv)	Maximal recovery 99.8% for Au(iii), Pt(iv), with 87.7% and then Pd(ii) 72.7% with 10 mg L^−1^ initial adsorbate concentration	19
Fe_3_O_4_	Hydrothermal	Biological application	Spherical Fe_3_O_4_ NPs possibility of using these NPs in biological applications	21
Fe_3_O_4_	Electrochemical deposition	Biomedical application	Can be used in biomedical applications	22
Magnetic iron oxide NPs	Microemulsion	(Phosphate removal)	Phosphate reduction was (>95%) in 5 min. and (100%) in 20 min	23
Magnetic iron oxide NPs	Laser pyrolysis	Biomedical	Increase in surface charge density	24
Fe_3_O_4_	Sonochemical method	MRI application	Exhibited high potential in the MRI application	25
Fe_3_O_4_	Microwave-assisted method	Advanced biomedical sensing application	Can be applied in MRI, hyperthermia, disease marker detection, and in targeted drug delivery	27
Magnetic iron oxide NPs	Flame spray pyrolysis	Biomedical application	Showed a successful validation *in vitro* biological evaluation for biomedical application	28
Fe_3_O_4_–Fe_2_O_3_	Chemical vapor deposition	Biological activity *in vivo*	A protective effect on (RBC, Hct, and Hb) can be seen with a dose of 6.75 mg Fe per kg and activates the regenerative response of bone marrow of laboratory animals	29

In fact, the co-precipitation method has always been considered the most common method due to its simplicity and the absence of hazardous materials. This method includes producing iron oxide particles by aging a stoichiometric mixture of ferrous and ferric salts in aqueous media. Actually, the particle shape, size, and composition depend on several factors, such as the Fe^3+^/Fe^2+^ ratio, temperature, pH solution, ionic strength of the media, and type of salts used.^30,31^ The chemical reactions involved for Fe_3_O_4_ information can be written as follows:Fe^2+^ + 2OH^−^ → Fe(OH)_2_Fe^3+^ + 3OH^−^ → Fe(OH)_3_Fe(OH)_2_ + 2Fe(OH)_3_ → Fe_3_O_4_ + 4H_2_O

A complete precipitation of Fe_3_O_4_ can be expected in the range (9–14) of pH with a molar ratio of 2 : 1 (Fe^3+^ : Fe^2+^) under a non-oxidizing oxygen-free environment. The γ-Fe_2_O_3_ can be obtained by oxidizing Fe_3_O_4_ in the presence of oxygen, as shown in the reaction below:Fe_3_O_4_ + 0.25O_2_ + 4.5H_2_O → 3Fe(OH)_3_2Fe(OH)_3_ → γ-Fe_2_O_3_ + 3H_2_O

**Fig. 2:**
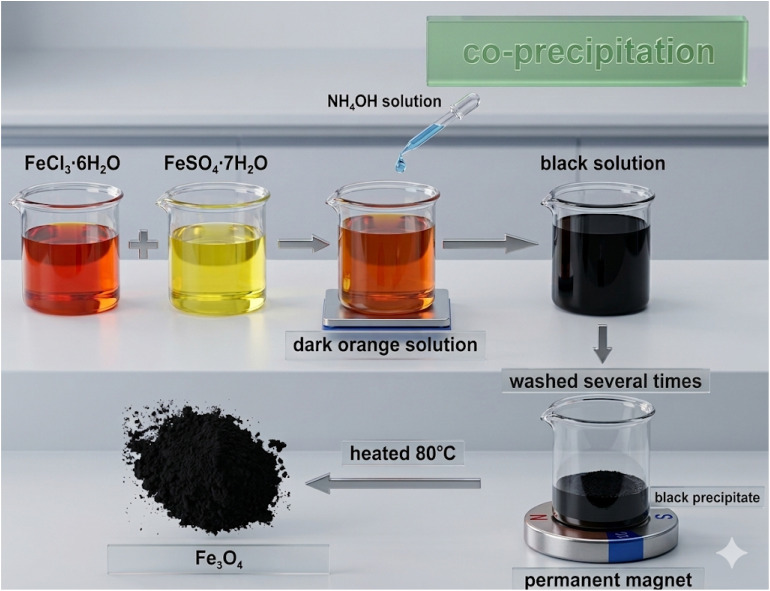
Procedure flow chart of nano magnetic Fe_3_O_4_ synthesis by co-precipitation method reproduced from ref. 34 with permission from Elsevier, Copyright © 2022. Modifications were made using Gemini AI-assisted tool.

Maintaining an oxygen-free environment by purging the solution with nitrogen is crucial, as it suppresses oxidation and promotes the formation of smaller, more uniform nanoparticles. Fig. 2 illustrates the co-precipitation synthesis route for Fe_3_O_4_ nanoparticles, in which FeSO_4_ and FeCl_3_ precursors are combined at a molar ratio of 1 : 2 to achieve controlled magnetite formation.^32,33^

The co-precipitation method consists of two steps: the first is the formation of small nuclei in the medium when the species concentration reaches the critical supersaturation, followed by the second step, a slow, controlled growth of the small nuclei, during which diffusion occurs from the solution to the crystal surface. It is very important to separate the nucleation and crystal growth steps to avoid a narrow particle size distribution after crystal growth.^35^ A comparison of the most important synthetic methods for magnetic iron oxide nanoparticles (MIONPs) is shown in Table 3.

**Table 3:** A comparison of the most important synthetic methods of MIONPs

Method	Advantages	Defects	Magnetic saturation value (emu g^−1^)
Laser pyrolysis	Exhibited no aggregation, small particle size	Very expensive, complicated	∼70
Microwave	Easy handling, short reaction time, higher performance	Not favorable for scale-up, high cost	40–100
Co-precipitation	Rapid, simple, easy to control particle size, can easily scale up	High agglomeration makes it difficult to control the shape and morphology of NPs	20–80
Thermal decomposition	Very high yield, high dispersion	Expensive, high temperature, long reaction time	<70
Hydrothermal	Easy to control particle size	Required high temperature and pressure	50–75

Polymer shields prevent MIONP oxidation and provide additional stability to the nanoparticles. Different strategies have been used to modify MIONPs with polymers, including post-synthesis and *in situ* coating methods. The post-synthesis coating can be achieved by chemical reactions or by the interaction of the polymer with the magnetic core, while *in situ* coating can be achieved by mini/micro-emulsion polymerization or a sol–gel process, without any degradation of the magnetic core.^36,37^ The stability gained from coating with inorganic materials is widely used, especially in biomedical applications, drug targeting, cell separation, oil recovery, and enzyme immobilization.^38,39^ The main inorganic compounds that are used in coating magnetic IONPs are: metals and metal oxides, noble metals, silica, carbon-based material, and sulfides.^35,38–42^

The most thermodynamically stable iron oxide phase in ambient circumstances is α-Fe_2_O_3_. Hematite solely contains Fe^3+^, in contrast to Fe_3_O_4_, which contains both Fe^2+^ and Fe^3+^, hence oxidation conditions and thermal treatment are essential during production. The α-Fe_2_O_3_ is very similar to magnetite synthesis, but with controlled oxidation:Fe^3+^ + OH^−^ → Fe(OH)_3_ → thermal treatment → α-Fe_2_O_3_

If starting from Fe_3_O_4_:

Fe_3_O_4_ → γ-Fe_2_O_3_ → α-Fe_2_O_3_ (at elevated temperature ∼500–800 °C). Phase transformation control is critical. Slow heating rate and sufficient annealing stabilize hematite. Comparison between synthesis of magnetite (Fe_3_O_4_) and hematite (α-Fe_2_O_3_) are listed in Table 4.

**Table 4:** Comparison between magnetite (Fe_3_O_4_) and hematite (α-Fe_2_O_3_) synthesis

Parameter	Magnetite (Fe_3_O_4_)	Hematite (α-Fe_2_O_3_)
Iron oxidation state	Fe^2+^ + Fe^3+^	Fe^3+^ only
Stability	Metastable	Thermodynamically stable
Magnetic behavior	Ferrimagnetic	Weakly ferromagnetic/antiferromagnetic
Typical synthesis	Co-precipitation	Hydrothermal + calcination

### 3.2 Greener synthesis of magnetic iron oxide NPs

**Fig. 3:**
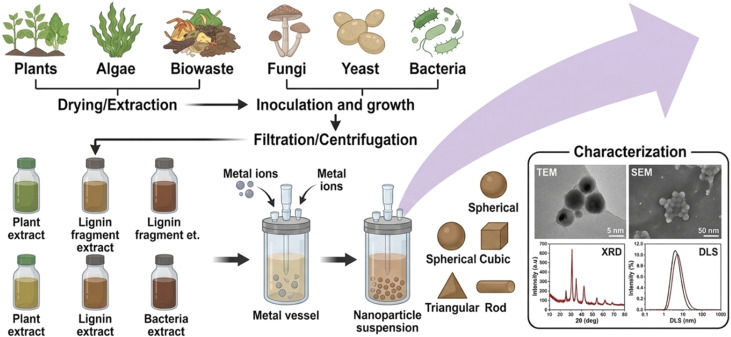
Green synthesis mechanism of magnetic iron oxide nanoparticles (IONPs) reproduced from ref. 50 with permission from MDPI, Copyright © 2023. Modifications were made using Gemini AI-assisted tool.

Eco-friendly synthesis strategies rely on green precursors, such as plant extracts and microorganisms (algae, bacteria, and fungi), which can be optimized to tailor nanoparticle morphology while maximizing the yield of magnetic iron oxide nanoparticles.^43^ It is an efficient method compared to other conventional methods.^44^ Al-Masri *et al.* have been synthesized Fe_3_O_4_ NPs by using leaves of plant (thyme and rosemary), which the extracts of both plants were been used to be mixed with Fe(ii) and Fe(iii) solutions at pH (10 to 11) followed by a centrifugation step at (4300 rpm for 20 min.) to collect the Fe_3_O_4_ NPs, attending with washing and drying steps, respectively.^45^ The most relevant advantages of the biological synthesis of magnetic IONPs are cost-effective, simple, fast, non-toxic to the environment, and an eco-friendly method.^46^ In terms of green synthesis advantages, the applications of green-synthesized magnetic IONPs have been found in numerous fields, such as biomedical (antimicrobial and anticancer) and wastewater treatment as adsorbents.^45,47–49^ The green process for producing magnetic iron oxide nanoparticles is shown in Fig. 3.

## 4 Modification of MIONPs surface nature

**Fig. 4:**
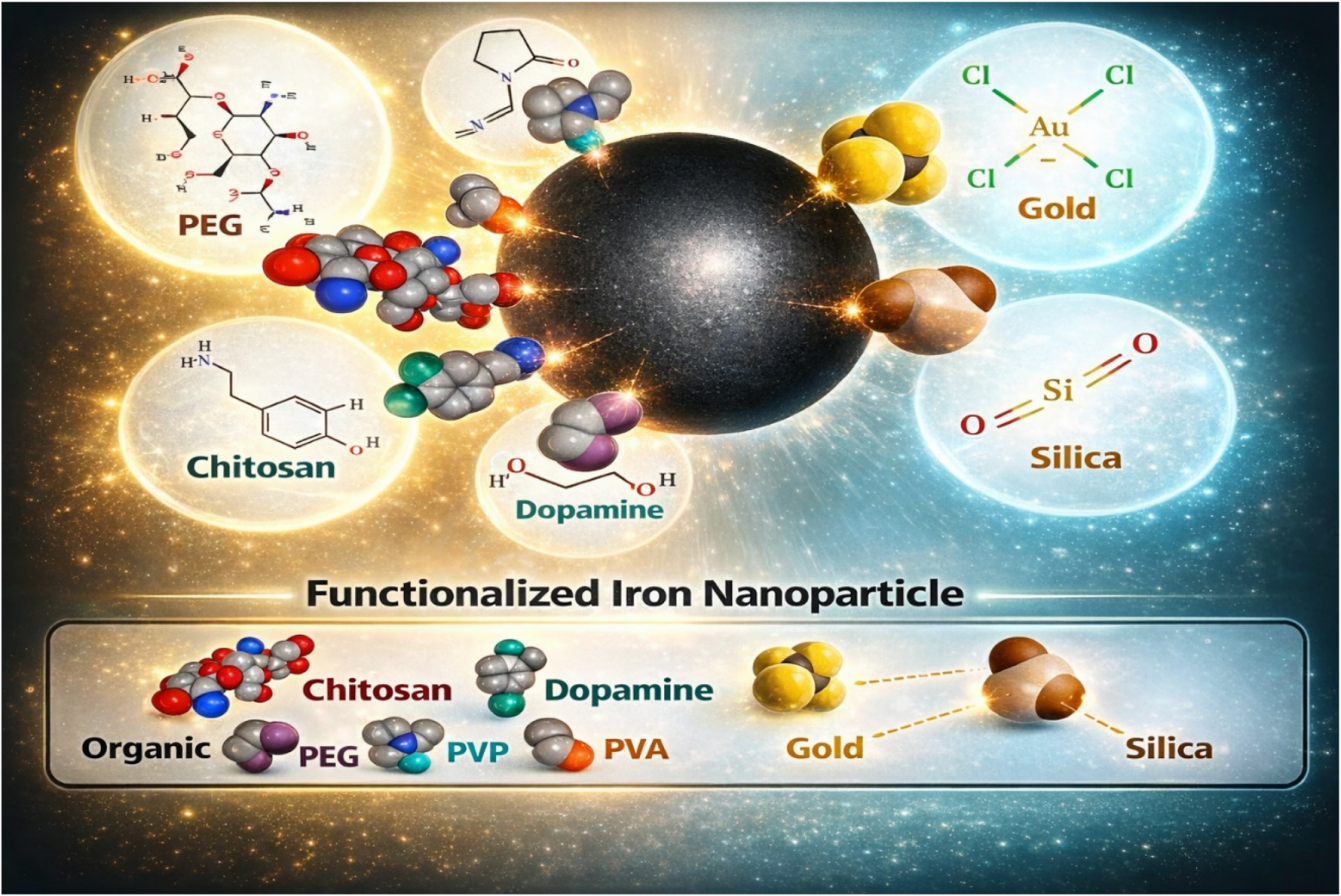
Various surface modifications of MIONPs reproduced from ref. 52 with permission from MDPI, Copyright © 2021. Modifications were made using Gemini AI-assisted tool.

The modification of magnetic nanoparticles can be achieved by adding organic or inorganic shells leading to extraordinary changes in the nanoparticles' morphology because of the additional layers, which provide an extra band of properties and, consequently, affect their performance and applications.^51^ Actually, the purpose behind modification is to overcome the agglomeration of MIONPs that happen after a period of time (due to the van der Waals forces, activation energy of the surface, and attractive magnetic forces between the nano particles), to get a biocompatibility for the magnetic IONPs, and to improve the physicochemical and mechanical properties, Fig. 4 shows various surface modification of MIONPs.^52^ The prevalent Surface modification methods for magnetic iron oxide nanoparticles are precipitation reaction method,^53^ electrostatic self-assembly method,^54^ sol–gel method,^55^ and Stöber method.^56^

Organic shell coatings can be made of a variety of materials, including natural and synthetic polymers such as starch, gelatin, chitosan, polyethyleneimine, poly(acrylic acid), poly(d,l-lactide) (PLA), polyethylene glycol (PEG), polystyrene, and polymethyl methacrylate (PMMA).^57–60^ These polymer-coated structures have been extensively employed in diverse applications, including catalysis,^61^ adsorption,^62^ magnetic resonance imaging (MRI),^63^ biosensing,^64^ and drug delivery systems.^65^ It is clearly evident in Fig. 4, strong magnetic dipole–dipole interactions and high surface energy of magnetic nanoparticles, like Fe_3_O_4_, encourage aggregation during membrane manufacturing. Furthermore, poor compatibility with hydrophobic membrane polymers like PVDF, PES, or PSf is caused by the excess of surface hydroxyl (–OH) groups. In order to control membrane microstructure, interfacial adhesion, and nanoparticle dispersion, surface modification is crucial. Co-precipitation-produced magnetic iron oxide nanoparticles, like Fe_3_O_4_, frequently have surface hydroxyl groups that encourage agglomeration because of magnetic dipole interactions. By adding functional groups (–NH_2_, –COOH) through surface modification with silane coupling agents (such as APTES), hydrophilicity is enhanced. Improve interfacial adhesion, decrease nanoparticle aggregation, increase compatibility with hydrophobic polymers (such as PVDF and PES), and have an impact on membrane shape through more uniform pore distribution and less macrovoid formation. Consequently, higher mechanical strength, increased water flux, and improved antifouling qualities.^66^ The surface characteristics of nanoparticles have a major impact on the dynamics of solvent–nonsolvent exchange during phase inversion. Unmodified Fe_3_O_4_ aggregates in the casting solution, causing structural flaws, pore blockage, and variations in local viscosity. Because modified Fe_3_O_4_ disperses evenly, serves as a nucleation site, and encourages controlled demixing, the membrane displays: reduced macrovoid collapse, enhanced porosity, better pore interconnectivity, and more consistent pore size distribution. Improved porosity architecture and well-developed finger-like structures result from modified nanoparticles' enhanced surface homogeneity and hydrophilic functional groups, which speed up water input during immersion.^67^

## 5 Comparison between conventional and sustainable manufacturing routes for magnetic iron oxide nanoparticles

Conventional manufacturing of magnetic iron oxide nanoparticles typically relies on high temperatures, toxic chemical reagents, and energy-intensive processes, raising concerns about environmental impact and occupational safety. In contrast, sustainable manufacturing approaches such as green synthesis using plant extracts or microbial routes operate under milder conditions and minimize hazardous by-products. These eco-friendly methods reduce energy consumption and chemical waste while improving biocompatibility and surface functionality of the nanoparticles. Moreover, green routes often enable better control over particle stability through naturally derived capping agents. Despite current challenges in scalability and reproducibility, sustainable synthesis presents a promising alternative to conventional methods for environmentally responsible nanomaterial production.^68^

For example, magnetic iron oxide nanoparticles synthesized *via* conventional co-precipitation commonly require strong bases (*e.g.*, NaOH or NH_4_OH), elevated temperatures, and synthetic surfactants to control particle growth, generating alkaline wastewater and secondary pollution. In contrast, green synthesis using plant extracts (such as tea leaves or neem) employs naturally occurring polyphenols as reducing and stabilizing agents under ambient conditions. This bio-assisted route significantly reduces energy demand and eliminates toxic reagents, while simultaneously imparting surface functional groups that enhance nanoparticle dispersion and environmental compatibility.^69^

## 6 Characterization of magnetic IONPs

**Fig. 5:**
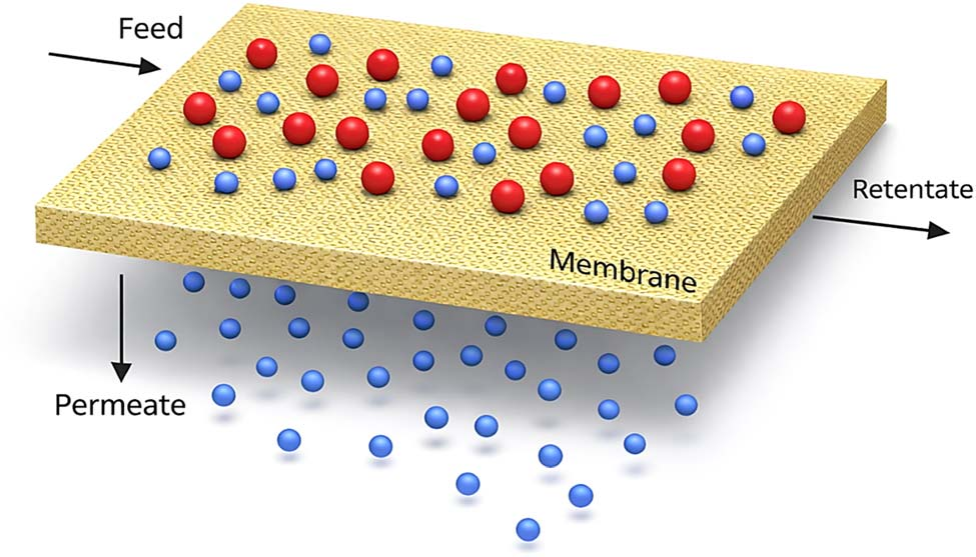
Membrane basics configuration reproduced from ref. 107 with permission from In Tech, Copyright © 2011. Modifications were made using Gemini AI-assisted tool.

Multiple techniques have been used to characterize the magnetic iron oxide nanoparticles, as shown in Fig. 5, which reveal their physical, chemical, and surface properties. These techniques can be classified into: microscopic, magnetometric, and spectroscopic techniques.^70^

### 6.1 Microscopic techniques

These techniques are commonly used to determine the shape, particle size, and morphology of magnetic IONPs, and this is achieved using high-resolution electron microscopy. Atomic force microscopy (AFM), transmission electron microscopy (TEM), and scanning electron microscopy (SEM) techniques are examples of this technology. Although SEM technique provides high-resolution images by the reflected electrons, TEM technique works with the detecting transmitted electrons which gives an information of the inner structure like crystal structure and the stress state for the magnetic IONPs, in addition, there is an atomic force microscopy (AFM) that also provides images of high resolution, three-dimensional topographic and gives a valuable information of physical and chemical properties.^71^

### 6.2 Magneto metric techniques

The most common techniques to measure the magnetic properties are vibrating sample magnetometry (VSM) and the superconducting quantum interference device (SQUID), and this is achieved by exposing the magnetic IONPs to a magnetic field H with a range of (−10 000 Oe to 10 000 Oe) for measuring coercive field, saturation magnetization, and remnant magnetization.^26,54^

### 6.3 Spectroscopic techniques

Many of these techniques can provide a clear picture of magnetic IONPs' properties; for example, the structure and functional groups can be obtained by Fourier Transform Infrared spectroscopy (FTIR), which is characterized by its speed, accuracy, and high precision. The FTIR basis work is summarized by the atomic vibrations of a molecule that absorb specific frequencies and energies of infrared radiation, and generally the absorbing light is in the region (2.5 µm–15 µm), corresponding to a wavenumber range (4000 cm^−1^ to 660 cm^−1^).^72^ In contrast, X-ray diffraction (XRD) is used to obtain information on the crystalline structure of IONPs. Furthermore, Dynamic Light Scattering (DLS) has been used to measure the hydrodynamic diameter of magnetic IONPs in the range of (30 nm–190 nm) with the aid of the Stokes–Einstein equation.^73^ The surface charge can be obtained by Zeta potential measurement.^74^ Also, there is thermogravimetric analysis (TGA) to determine the optimal reaction temperature and thermal stability of magnetic IONPs.^75^

## 7 Adsorption powerful of magnetic IONPs

In the recent years, nanotechnology have been taken an important role as an adsorbent and it can be used in diverse applications, which is because of its magnificent characteristics (high surface area and the tunable surface chemistry) as compared to other treatment techniques, and consequently an enhancement in efficient removal of pollutants as well as easy separation from the solutions.^76–78^ Among several kinds of nanomaterials, magnetic IONPs have proven their impact in wastewater treatment applications.^33,41,42^ The adsorption capacity of MIONPs is very high, reaching up to 99.8% for adsorbing Al ions from polluted aqueous solutions, and only 3 minutes were required to achieve complete removal of Al ions.^79^ Rashid *et al.* studied arsenic removal using prepared nanoscale zero-valent iron (NZVI), achieving a removal efficiency of 99.57%. In comparison, a model predicted 99.82% removal efficiency, which is very close to the experimental result.^80^ In contrast, the removal efficiency was 84.72% for Cu(ii) adsorption using APTMS-BCAD-modified Fe_3_O_4_, with an adsorption capacity of 43.67 mg g^−1^, in real water samples and CRM. The study demonstrated that complexes with oxygen-containing functional groups and amine groups form a surface structure when Cu(ii) is adsorbed on the nanocomposite surface, and this improves the adsorption capability because of the presence of free amine groups as the pH rises.^81^ Quach and Doan prepared superparamagnetic iron oxide nanoparticles (SPIONs) within particle size around 13.6 nm modified with polyvinyl alcohol, graphene oxide, and chitosan, to be used in methylene blue (MB) adsorption, where the study achieved approximately 87% entrapment efficiency at pH 7 after 13 days, while (36.385 ± 0.095 mg g^−1^) was the highest loading amount, so, the results indicated very good impacts toward dyes adsorption.^18^ Au(iii), Pt(iv), and Pd(ii) were recovered from aqueous solutions by Amuanyena *et al.* The adsorption was investigated at room temperature (25 °C). The results showed that the modified iron oxide nanoparticles were successful in the selective recovery of precious metal ions, with maximum recoveries of 99.8%, 87.7%, and 72.7% for Au(iii), Pt(iv), and Pd(ii) at pH 2.5, 10 mg L^−1^ initial adsorbate concentration for 120 minutes of agitation time and 0.065 g adsorbate dosage.^19^ Cusioli *et al.* developed modified magnetic iron oxide nanoparticles (MOM-Fe_3_O_4_) by functionalizing the residues of *Moringa oleifera* Lam. Seed husks on the surface of iron nanoparticles, to be applied in the removal of metformin from contaminated water, the study achieved 93.9% reduction after 720 minutes in metformin content with 65.01 mg g^−1^ adsorption capacity, so the study indicated that (MOM-Fe_3_O_4_) as a low-cost adsorbent particle was successful for the removal of pharmaceuticals like metformin.^82^Table 5 summarizes the most important studies published in the last few years on adsorption processes using magnetic nanoparticles to remove various pollutants.

**Table 5:** Summary on the adsorption of pollutants by MIONPs

Magnetic nanoparticles	Pollutant	Adsorption capacity (mg g^−1^)	Performance	Ref.
(AC/Fe_3_O_4_) pomace waste precursor (PP) nanocomposite	COD, BOD_5_, turbidity, TDS	962.31 mg g^−1^	(AC/Fe_3_O_4_) PP nanocomposite was effective in treating the tannery wastewater	86
Fe_3_O_4_ NPs@nanoclay composite	Ketoprofen (KP), naproxen (NX)	—	Removing (>90%) of the two drugs	87
Polypyrrole-magnetic oxide-graphene (FOPYGO) nanocomposite	Methylene blue dye, orang G	30.07 mg g^−1^ (for methylene blue dye), (and orange G dye)	(96.8%) adsorption efficiency for methylene blue and (20%) for orange G dyes	88
(Fe_3_O_4_ NRs) in a cellulose nanocrystal (CNC) polymer hydrogel [CNC-g-PAA/Qp4vp (CPqP)]	Arsenic As(iii)	—	A great potential as an adsorbent for As(iii) removal	89
CuFe_2_O_4_/sepiolite/graphene oxide GO (CFSG)	Pb(ii), Cd(ii)	238.1 mg g^−1^ (for Pb(ii)), 14.97 mg g^−1^ (for Cd(ii))	Adsorption Pb(ii) adsorption Cd(ii). CuFe_2_O_4_ facilitates the adsorption process	90
Fe_3_O_4_–MnO_2_	As(iii)	81.16 mg g^−1^	96% removal of As(iii), and the regenerated Fe_3_O_4_–MnO_2_ was able to remove 88% of As(iii)	91
Fe_3_O_4_ NPs	Congo red (CR)	780 mg g^−1^	Efficient adsorbent for the CR dye	92
CuFe_2_O_4_-NPs (CFN)	4-Nitrophenol (4-NP), indigo carmine (IC)	57.4 mg g^−1^	80% removal efficiency after five cycles for the (IC) dye, as well as CFN demonstrated a reductive elimination of 4-NP	93
Fe_3_O_4_/alginate-bentonite core and chitosan shell layer (mAB@Cs)	Organic ciprofloxacin (CPX), (Cu^2+^, Cd^2+^, Co^2+^, Ni^2+^, Pb^2+^, Zn^2+^, and Hg^2+^) potassium K^+^, orthophosphate phosphorous P–PO_4_^3−^, BOD, and COD	264.7 mg g^−1^	High removal of heavy metals 99.86%. The removal was 46%, 90%, 84%, and 64% for potassium K^+^, orthophosphate phosphorous P–PO_4_^3−^, BOD, and COD, respectively. Also, a high inactivation rate of *E. coli*, reaching 96%	94
Graphene oxide-supported cobalt–iron oxide (GO/Co–Fe) magnetic nanocomposite	Tetracycline (TC), chlortetracycline (CTC), oxytetracycline (OTC), and doxycycline (DTC)	64.1 mg g^−1^ (for TC), 71.43 mg g^−1^ (for CTC), 72.46 mg g^−1^ (for OTC), 99.01 mg g^−1^ (for DTC)	A high removal of 94.32% (CTC), 94.1% (TC), 96.94% (DTC), and 94.22% (OTC)	95
Biocarbon/aluminum hydroxide nanocomposite [BC/Al (OH)_3_-Fe_3_O_4_-NC]	Cobalt Co(ii), cadmium Cd(ii), strontium Sr(ii)	113.53 mg g^−1^ for Co(ii), 102.86 mg g^−1^, for Cd(ii), 89.97 mg g^−1^ for Sr(ii)	A 99.65%, 99.45%, and 99.78% for Cd(ii), Co(ii), and Sr(ii), respectively	96
Fe_2_O_3_	Levofloxacin	33.1 mg g^−1^	Removal of levofloxacin was exothermic & spontaneous (according to thermodynamic parameters)	97

The dispersion behavior, magnetic responsiveness, surface activity, and overall impact on membrane performance of magnetic iron oxide nanoparticles (IONPs), especially Fe_3_O_4_ and γ-Fe_3_O_4_, are all determined by their physicochemical characteristics. Thus, thorough characterization with alternative analytical methods is crucial. The most popular characterization techniques are included in this part, along with an analysis of their importance based on published research. The crystalline phase, structural purity, and crystallite size of magnetic IONPs are frequently ascertained by X-ray diffraction (XRD). The typical inverse spinel cubic structure of magnetite (Fe_3_O_4_) has diffraction peaks that correspond to the (220), (311), (400), (422), (511), and (440) planes. The Scherrer equation is commonly used to estimate the average crystallite size. According to findings in the literature, IONPs with crystallite sizes less than about 20 nm frequently display superparamagnetic behavior, which is ideal for membrane separation applications since it eliminates magnetic remanence and aggregation when an external magnetic field is removed.^83^ FTIR spectroscopy is used to verify chemical interactions between IONPs and the polymer matrix and to identify surface functional groups. Iron oxide production is confirmed by the Fe–O stretching vibration of magnetite, which usually appears between 550 and 600 cm^−1^. Surface-modified IONPs exhibit extra peaks corresponding to hydroxyl, carboxyl, or silane groups, which improve compatibility with polymeric membranes. For confirming chemical bonding or intermolecular interactions between IONPs and functional groups in polymers like PES, PVDF, or PSf, FTIR measurement is especially crucial in membrane systems.^84^ Membrane surface morphology, pore structure, and nanoparticle dispersion are investigated using scanning electron microscopy (SEM). IONPs must be distributed uniformly across the membrane matrix to prevent agglomeration, which can impair mechanical stability and permeability. On the other hand, nanoparticle size, shape, and aggregation state can be directly observed at the nanoscale using transmission electron microscopy (TEM). Several investigations published in desalination have shown that while excessive loading causes particle clustering and pore blockage, well-dispersed Fe_3_O_4_ nanoparticles improve membrane hydrophilicity and antifouling capabilities.^85^

## 8 Fundamentals of membrane technology

Membrane technology can be considered the preferred technology for water treatment, due to its remarkable features of high separation efficiency, lower energy consumption, the ability to remove various contaminants, and its continuous operating mode.^98,99^ The membrane essentially acts as a barrier separating two phases and controls the transport of different components, as seen in Fig. 5. Membrane categories under different classifications according to membrane geometry, material, preparation techniques, and morphology. Membrane geometric shapes can take flat-sheet, hollow-fiber, or tubular forms. Membranes can be composed of organic materials (polymers), inorganic materials (ceramics), or both, which is called a mixed matrix membrane (MMM). Polymeric membranes are known for their physical strength, flexibility, and chemical stability. However, polymeric membranes suffer from low tolerance to elevated temperatures and a corrosive environment, as well as to organic solvents.^100^ The most prevalent polymeric material known for its chemical and thermal stability is polysulfone (PSF).^101^ A wide range of polymers is also used in membrane fabrication, including polyethersulfone (PES), polyvinylidene fluoride (PVDF), polyvinyl chloride (PVC), polyacrylonitrile (PAN), polyvinyl alcohol (PVA), polyamide (PA), polypropylene (PP), and polyethylene (PE).^102^ Ceramic membranes are widely used in separation processes due to their compatibility with strong media such as acids and strong solvents, as well as extreme conditions such as high temperature and pressure.^103^ On the other hand, ceramic membranes are recognized as excessively brittle and have high production costs, which can limit their application in large-scale industries.^104^ Actually, Cellulose acetate (CA), cellulose, and ethyl cellulose were the materials of the first commercial membranes.^105^ Membranes are usually synthesized by using the phase-inversion method or mechanical stretching for polymeric materials, and by the colloidal suspension method for metal hydroxide deposition on porous supports for ceramic materials.^106^

### 8.1 Advances in mixed matrix membranes (MMMs) fabrication techniques

The combination of polymeric membranes and inorganic or organic fillers in MMMs has contributed to an additional improvement in membrane separation performance. Because of this superior mixture, a range of fabulous properties have been observed in the MMMs, such as increased polymer flexibility and the superb thermal, chemical, and mechanical properties of fillers like magnetic iron oxide nanoparticles (MIONPs) or zeolites. There are various characterization techniques for MMMs in order to characterize the morphology and structure, which are described in Table 6 (ref. 108 and 109) and study the performance of the membrane (fouling with (FRR) parameter, permeability with (PWF) parameter, and selectivity). Actually, the presence of magnetic iron oxide nanoparticles (MIONPs) can improve the membrane's permeability, selectivity, and durability, making it more effective in various applications such as water treatment and gas separation.^110,111^

**Table 6:** Characterization techniques of magnetic (MMMs)

Technique	Outcome
Vibrating sample magnetometry (VSM)	Saturation magnetization, coercivity
X-ray diffraction (XRD)	Crestal lattice data, average crestal size
Dynamic light scattering (DLS)	Hydrodynamic particle size distribution, polydispersity index
Fourier-transform infrared spectroscopy (FTIR)	Existence of organic and inorganic components (functional groups)
Atomic force microscopy (AFM)	Poor size, shape, surface topology
(SEM)	Images with high resolution for analyzing the magnetic MMMs surface topography
(TEM)	Images with high resolution showing magnetic MMMs' poor size distribution and shape

There are several fabrication techniques for mixed matrix membranes (MMMs), such as phase inversion,^110^ electrospinning,^112^ and self-assembly layer-by-layer (LBL).

## 9 Fabrication methods of magnetic mixed matrix membranes (MMMs)

**Fig. 6:**
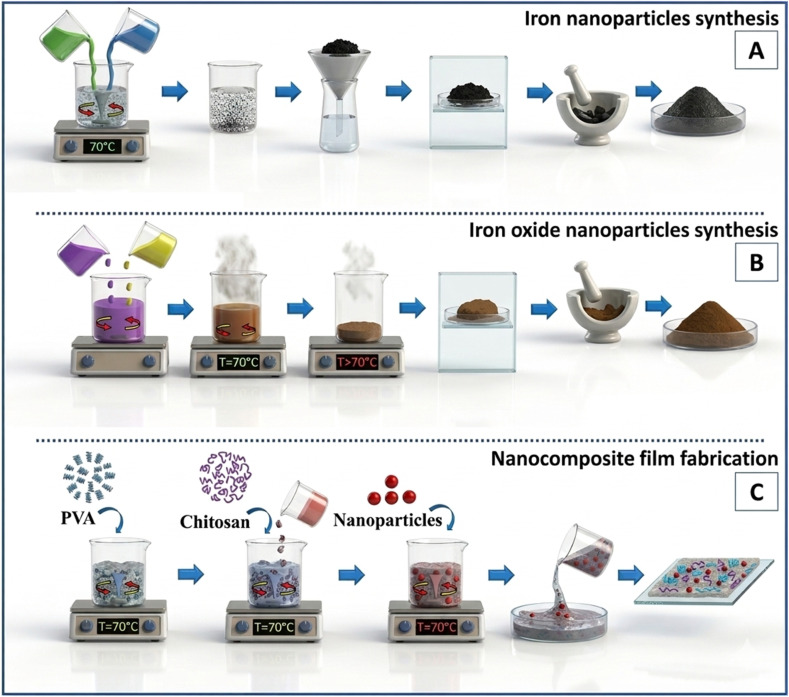
Fabrication of magnetic iron oxide by (A) chemical reduction, (B) sol–gel methods and (C) nanocomposite MMMs reproduced from ref. 115 with permission from Elsevier, Copyright © 2018. Modifications were made using Gemini AI-assisted tool.

The advancement of mixed matrix membranes has been significantly enhanced by utilizing magnetic IONPs, owing to the remarkable properties of magnetic materials. The commonly fabricated MMMs methods are the sol–gel method,^113,114^ solution mixing, and *in situ* polymerization. Synthesis of magnetic MMMs is achieved by using organic monomers or polymers with the inorganic magnetic NP precursor in a solution, and during the hydrolysis process, the inorganic magnetic precursor will generate a uniform distribution of magnetic NPs, and consequently, a polymer matrix will be created, as shown in Fig. 6.^115^

While solution mixing or blending method is almost identical to the regular manufacture of (MMMs), which involves the dissolving of polymer in solvent and then stirring, followed by the addition of inorganic particles to the polymer solution, as well as, the inorganic fillers can also be added and dispersed into the solvent before the addition of polymer and stirred individually apart from the step of dissolving polymer into the solvent, after that, the particle suspension is integrated into the polymeric solution, followed by a casting with blade for the resultant mixture. Finally, it was exposed to an allowable solvent evaporation at a specified temperature.^116^ So, magnetic (MMMs) can be fabricated using magnetic IONPs such as Fe_3_O_4_.^117^

In the *in situ* polymerization fabrication method, organic monomers are incorporated during the blending of filler materials prior to the polymerization reaction; as a result, a functional group is attached to the filler particle surface, thereby enhancing the polymerization of monomers on that surface. A covalent bond can form between nanoparticle fillers with functional groups and the polymer chains, thereby improving the compatibility between the polymer and particle phases.^118,119^

To obtain a uniform distribution of magnetic IONPs, the membrane is exposed to an external magnetic field, which creates a magnetic channel within the membrane that enhances the dispersion of the magnetic IONPs within the magnetic membrane and, consequently, improves membrane performance, such as permeability and selectivity. Also, the selection of fillers and their physicochemical compatibility with the membrane polymer type is very important for obtaining substantial magnetic MMMs.

## 10 IONPs incorporated into mixed matrix membranes in wastewater treatment

The role of magnetic IONPs has been very effective and crucial in improving the MMMs performance, including the permeability and the selectivity, which drive it to be used in gas separation^120^ and diverse aquatic separation processes such as wastewater treatment,^121^ desalination,^122^ dye removal,^123^ and heavy metal removal.^124^ The modified properties of magnetic (MMMs), especially membrane antifouling, can limit operational expenses and increase the durability of the membrane, as seen in Zhang *et al.*, where a polyethersulfone (PES) membrane incorporated with Fe_3_O_4_ NPs was prepared by the casting method. The study found that there was an enhancement in antifouling capability and an increase in membrane permeability and rejection performance to 913.39 LMH bar^−1^ and 93%, respectively, owing to the tiny pores formed on the membrane surface, which facilitated solvent diffusion during phase separation.^117^ In another study, the results showed a high flux value of 16.35 L m^−2^ h^−1^ when 0.01% of magnetic IONPs (Fe_3_O_4_) is added. A flux value of 2.81 L m^−2^ h^−1^ was observed for the bare membrane, and the salt rejection increased from 60% to 90% for the magnetic membrane.

Furthermore, it has been observed that magnetic IONPs have a positive charge. Still, at high concentration levels of (Fe_3_O_4_), these particles tended to agglomerate, and their surface charge was noticed to be a negative charge.^110^ Suryanto *et al.* have been studied dye (congo red and acid orange) and bacterial removal by utilizing pineapple biowaste extraction to fabricate a bacterial cellulose acetate-based membrane incorporated magnetic nanoparticles(Fe_3_O_4_) of (0.25, 0.5, 0.75, and 1 wt%), the study revealed that there is been an increasing in tensile strength to 73%, and the work has been successful in removing bacteria from river wastewater to 67.4% and very good in improving the adsorption of dyes.^125^

Esmail *et al.* have been utilized (0.2 wt%) magnetic (Fe_2_O_3_) NPs in (poly vinylidene fluoride) PVDF to fabricate a magnetic MMM for oily wastewater treatment, and the study revealed a magnificent effect of iron oxide NPs on MMMs properties, which exhibited 46 L m^−2^ h^−1^ of water flux (20% higher than neat PVDF), while for oil rejection was (more than 94%).^126^ So, incorporating a small loading (0.2 wt%) of magnetic iron oxide (Fe_2_O_3_) nanoparticles into PVDF membranes significantly enhances mixed-matrix membrane performance for oily wastewater treatment, improving water permeability without compromising separation efficiency.

Functional membranes' wettability and polarity affect water movement, fouling resistance, and pollutant rejection, all of which are important factors in water treatment applications. Higher water flux results from membranes with hydrophilic surfaces because they assist water adsorption and encourage capillary flow through the pores. Hydrophobic surfaces, on the other hand, may increase fouling tendencies and restrict water permeability. Similar to this, the membrane's surface polarity controls how it interacts with solutes; polar or charged surfaces can improve the rejection of dyes, ionic pollutants, and other polar pollutants through hydrogen bonding and electrostatic interactions. By functionalizing membranes with magnetic nanoparticles or other nanomaterials, surface wettability and polarity can be changed, increasing water permeability and decreasing fouling while preserving or improving selectivity. Therefore, creating high-performance membranes for water purification and other separation processes requires optimizing wettability and polarity in addition to incorporating nanoparticles. By encouraging water adsorption and facilitating capillary flow through the membrane pores, hydrophilic surfaces improve water flux. Surface polarity influences rejection efficiency by affecting how the membrane interacts with solutes, such as dyes, heavy metals, and other impurities. By functionalizing membranes with magnetic nanoparticles or other nanomaterials, surface wettability and polarity can be changed, improving water permeability and antifouling characteristics without sacrificing selectivity.^127–131^

The nanohybrid materials, such as graphene oxide nanosheet (GO) with iron oxide NPs, were used to synthesize a polyether sulfone (PES)-based (UF) mixed matrix membrane (MMM) (GO/Fe_3_O_4_/PES). The best performance of this membrane was observed at a permeance of 252 L m^−2^ h^−1^ bar^−1^ and 53.9° of water contact angle (WCA). Also, the pure water flux (PWF) increased up to 76.2%, and (WCA)decreased by 35% if compared with the neat PES membrane. Furthermore, (MMM)(GO/Fe_3_O_4_/PES) showed high rejection against bovine serum albumin (BSA) of around 92% and 87.9% for the flux recovery ratio (FBR).^132^ Hosseini *et al.* have been working on the fabrication of mixed matrix PES-based nano-filtration membrane integrated with magnetic (iron oxide-polyvinylpyrrolidone) composite nanoparticles for separation processes of salt rejection and BSA solution filtration, which were enhanced (up to 0.1% of Fe_3_O_4_/PVP) in the membrane body and then reduced with a higher NPs ratio. And this is to overcome the agglomeration of the nanocomposite that may occur at high concentration and reduce the performance of the magnetic MMMs. The water flux showed 9.96 L m^−2^ h^−1^ and 3.14 L m^−2^ h^−1^ for the membrane with and without magnetic nanocomposite, respectively. Also, the measured FRR was approximately 46.2% for the bare membrane, and with the range (69,9–89.5%) for the nanocomposite membrane. Finally, the contact angle has decreased from 65.18° for the bare membrane to 50.5° for the nanocomposite membrane.^133^Table 7 presents the most important studies published in the past few years on employing magnetic iron oxide nanoparticles in MMMs for wastewater treatment.

**Table 7:** Summary of MMMS incorporated magnetic IONPs for wastewater treatment

Membrane	Pollutant	Rejection%	Performance	Ref.
PES/Fe_3_O_4_	Bovine serum albumin (BSA)	80.43% for (50–100) nm & 77.65% for (<5 µm)	Hollow fiber membrane incorporation of 50 nm (Fe_3_O_4_) NPs to get a contact angle (75.77°), PWF (110.42 L m^−2^ h ^−1^ bar^−1^) for (50–100) nm PWF and (91.54 L m^−2^ h^−1^ bar^−1^) for (<5 µm)	134
Fe_2_O_3_/PES/CA	Fluoride	—	Fluoride removal of (70.3%) for a single ultrafiltration run and (156 L m^−2^ h^−1^) for PWF	135
PES/BiFeO_3_	Congo red dye	—	Maximal permeate flux (63.5 LMH) and (97.974%) dye removal at 0.25 wt% NPs & pressure of 2.57 bar	136
PVA/nano CoFe_2_O_4_	Congo red (CR) and malachite green oxalate (MG)	—	Membranes to reach 97.5% for (MG) dye while it was 92% for (CR) dye. (FT-IR), and (XRD)	137
PVA/nano NiFe_2_O_4_				
PVA/nano MnFe_2_O_4_				
f-Fe_3_O_4_/polysulfone (PSF)	Erichrome black-T, alizarin red	84.2%	A removal of erichrome black-T 94% and 99.6% for alizarin red. Permanence results are about 400 L m^−2^ h^−1^ with an FRR of 91.3%	138
FeO/PES	Salt solutions. CaCl_2_, MgCl_2_, C_6_H_5_Na_3_O_7_, Na_2_SO_4_, NaCl	>90%	Good flux (27.46 to 36.85 L m^−2^ h^−1^), and surface charge (−6.27 to −14.21 mC m^−2^)	139
Polysulfone (PsF)/magnetic NPs	Oil	∼99	Enhanced the pure water permeability to (∼200 L m^−2^)	140
Metformine-modified silica/Fe_3_O_4_	Copper	—	The removal of copper was 92%	141
Electrospun PES/Fe_3_O_4_	Oil	—	The oil elimination was 94.01% and water flux recovery 79.5%	142
PES/Cu@Fe_3_O_4_	(BSA) and (MB)	Up to 90% for (BSA) and (MB)	About (36%) an enhancement in thermal stability and mechanical strength in comparison with the bare PES membrane	143
Fe_3_O_4_/SiO_2_/KCC-1/GMSI/carnosine NPs/PES	Oil	—	PWF was 93.42 L m^−2^ h^−1^ with flux recovery ratio of 95.5%	144
Zero nano-valent iron (ZNVI)/polyamide thin film PA (TF)	Salts, methylene blue (MB), methyl orange (MO), congo red (CR)	98.6% for salt rejection	Pure water flux for (35 L m^−2^ h^−1^) at 15 bar, rejection (98.6%), and the flux recovery ratio (FRR) (87%)	145
Graphene oxide/iron oxide/poly-ethersulfone (GO/Fe_3_O_4_/PES)	Bovine serum albumin (BSA)	92% BSA rejection	PWF increased by 76.2%, flux recovery ratio of about 87.9%, while the total fouling was 41.5%	146
Polyethersulfone/TiO_2_–BiFeO_3_ (PES/TBF)	Congo red (CR)	94.84% (CR rejection)	PWF was 55 kg m^−2^ h^−1^ (PES/TBF) membrane	147
PES/Fe_3_O_4_/graphene oxide (BFGO) nanocomposite	Heavy metals (As, Cr, Cu, Ni, Zn, Co, Fe, Mn, Ti, and Be)	The removal was: (90.79%), Cr (91.77%), Cu (95.74%), Ni (91.78%), Zn (92.77%), Co (91.74%), Fe (90.75%), Mn (93.79%), Ti (91.79%), and (93.77%) Be	PWF increased to 408.1 L m^−2^ h^−1^ (BFGO membrane), fouling resistance rate reached 84.8%	148
PSf/TiO_2_–NiFe_2_O_4_@SiO_2_ nanocomposite	Sulfonamide antibiotic (SAM)	SAM removal efficiency was 85% in the dark and 95% under UV irradiation	The FRR increased to 95% permeate flux reached 72 L m^−2^ h^−1^ (under UV light). The reversible and irreversible fouling were ∼76% and ∼83% under UV irradiation	149
PES/biochar (BC), PES/biochar-Fe_3_O_4_ (FBC)	As(v), Cr	The removal was 99.9% for As and 99.8% for Cr	PWF increased to 359.4 L m^−2^ h^−1^	150

These improvements include notable reductions in contact angle, increased pure water flux (PWF), enhanced pollutant rejection, and higher flux recovery ratio (FRR). For instance, Suter *et al.* reported swelling ratios of 76.87 g g^−1^ and 58.23 g g^−1^ for pristine CNCs and N6 membranes, respectively. Upon incorporation of Fe_3_O_4_ nanoparticles, the swelling ratio increased to 109.54 g g^−1^ for CNCs/N6@Fe_3_O_4_ nanocomposites without chitosan and further to approximately 168.24 g g^−1^ in the presence of chitosan. This substantial increase highlights the synergistic role of magnetic IONPs, attributed to their high specific surface area and pore-forming capability, which collectively enhance membrane hydrophilicity and water uptake.^151^ Overall, Table 8 clearly demonstrates the superior performance of MMMs containing magnetic IONPs relative to their non-magnetic counterparts.

**Table 8:** The performance of MMMs with and without magnetic IONPs

Membrane	Pollutant	Rejection% or removal efficiency%	Performance (without magnetic IONPs)	Performance (with magnetic IONPs)	Ref.
Fe_3_O_4_/SiO_2_/KCC-1/GMSI/carnosine NPs/PES	Oil	—	Contact angle ∼76° at 0.1 wt% magnetic NPs	Magnetic NPs of 0.1 wt% reduced membrane contact angle from to ∼60.3°, PWF was 93.42 L m^−2^ h^−1^ with flux recovery ratio of 95.5%. The NPs were characterized by (FE-SEM) and (AFM)	144
Zero nano-valent iron (ZNVI)/polyamide thin film PA (TF)	Salts, methylene blue (MB), methyl orange (MO), congo red (CR), and malachite green oxalate (MG)	98.6% for salt rejection	Pure water flux (22 L m^−2^ h^−1^) at 15 bar, rejection 88.5%, and the flux recovery ratio (FRR) (78%)	Pure water flux for (TMC) ZNVI/PA(TF) NCs membrane; (35 L m^−2^ h^−1^) at 15 bar, rejection (98.6%), and the flux recovery ratio (FRR) (87%). The membrane and NPs were characterized by contact angle, mechanical testing, BET, FTIR, SEM, XRD, TEM, zeta potential, and EDX.	145
Graphene oxide/iron oxide/poly-ethersulfone (GO/Fe_3_O_4_/PES)	Bovine serum albumin (BSA)	92% BSA rejection	The total fouling was 84.35%, contact angle 82.9°	The contact angle of the (GO/Fe_3_O_4_/PES) membrane decreased by 35%, and its PWF increased by 76.2% in comparison with the neat PES membrane. The best performance for (GO/Fe_3_O_4_/PES) membrane was with a permeance of 252 L m^−2^ h^−1^ bar, contact angle (53.9°), and the flux recovery ratio of about 87.9%, while the total fouling was 41.5%	146
Polyethersulfone/TiO_2_–BiFeO_3_ (PES/TBF)	Congo red (CR)	94.84% (CR rejection)	PWF (10 kg m^−2^ h^−1^) (for pure PES membrane)	PWF was 55 kg m^−2^ h^−1^ (for PES/TBF), with wettability and porosity enhancing 25% and 35%, respectively. TEM, FESEM, FTIR, porosity studies, and contact angle have characterized the (PES/TBF) membrane	147
PES/Fe_3_O_4_/graphene oxide (BFGO) nanocomposite	Heavy metals (As, Cr, Cu, Ni, Zn, Co, Fe, Mn, Ti, and Be)	The removal efficiencies were: As (90.79%), Cr (91.77%), Cu (95.74%), Ni (91.78%), Zn (92.77%), Co (91.74%), Fe (90.75%), Mn (93.79%), Ti (91.79%), and Be (93.77%)	PWF was approximately 240.3 L m^−2^ h^−1^ (pure PES membrane), the contact angle was ∼70.7°	At the addition (1.5wt%), PWF increased to 408.1 L m^−2^ h^−1^ (BFGO membrane), while the contact angle decreased to 48.2°, the maximum fouling resistance rate reached 84.8%	148
PSf/TiO_2_–NiFe_2_O_4_@SiO_2_ nanocomposite	Sulfonamide antibiotic (SAM)	SAM removal efficiency was 85% in the dark and 95% under UV irradiation	The FRR was 65% (for neat PSF membrane). The permeate flux was 25 L m^−2^ h^−1^ for the neat PSf membrane	The FRR increased to 95% under UV light (for the composite membrane), while the permeate flux reached 72L m^−2^ h^−1^ (under UV light). The reversible and irreversible fouling were ∼76% and ∼83% under UV irradiation	149
PES/biochar (BC), PES/biochar-Fe_3_O_4_ (FBC)	As(v), Cr	The removal effectiveness was 99.9% for As and 99.8% for Cr	The PWF was 239.9 L m^−2^ h^−1^ (for PES/BC) membrane, the contact angle was 70.7°	The PWF increased to 359.4 L m^−2^ h^−1^ (for PES/FBC) membrane, and the contact angle decreased to 51.4° for FBC MMMs at (pH 10)	150

## 11 Mechanism of pollutant removal in magnetic MMMs

**Fig. 7:**
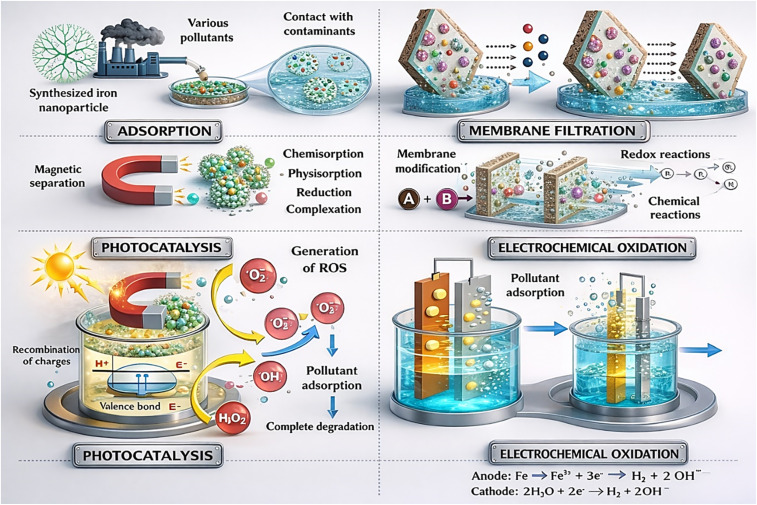
The mechanism of magnetic mixed matrix membranes (MMMs) reproduced from ref. 155 with permission from Elsevier, Copyright © 2025. Modifications were made using Gemini AI-assisted tool.

**Fig. 8:**
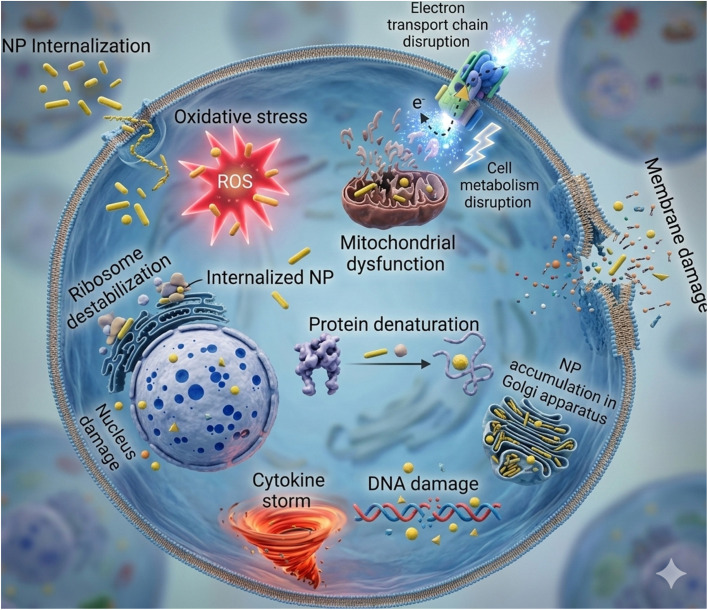
Effect of NPs on cell destruction reproduced from ref. 160 with permission from the Royal Society of Chemistry, Copyright © 2023. Modifications were made using Gemini AI-assisted tool.

The separation process in membrane technology usually depends on the solution-diffusion mechanism, which assumes that each permeating molecule is sorbed at one membrane interface, transported across the membrane by diffusion through the polymeric chains, and desorbed at the other interface. Pollutant removal in magnetic mixed matrix membranes (MMMs) occurs through a synergistic combination of size exclusion, adsorption, and electrostatic interactions. The embedded magnetic nanoparticles introduce additional active sites that enhance the adsorption of organic pollutants and heavy metals *via* surface complexation and hydrogen bonding. Improved membrane hydrophilicity and pore connectivity facilitate higher water permeability while maintaining effective rejection. Moreover, magnetic responsiveness enables external field-assisted fouling mitigation and, in some cases, *in situ* regeneration. Collectively, these mechanisms result in enhanced separation efficiency and antifouling performance compared with conventional MMMs.^152^ However, when magnetic nanomaterials are incorporated into the membrane structure, the mechanism changes to adsorption.^153,154^ The role of magnetic IONPs can be considered as adsorbent materials for the pollutant because of the electrochemical oxidation and generation of the reactive oxygen species (ROS), like ˙O_2_^2−^, ˙O_2_^−^, ﮲OH, and H_2_O_2_ as shown in Fig. 7.^155^ These oxidizing agents have the ability to perform the function of photocatalysts, which can effectively degrade the pollutants.^156^Fig. 8 illustrates the mechanism of magnetic mixed matrix membranes (MMMs).

## 12 Toxicity of magnetic IONPs

The ability of magnetic nanoparticles (MNPs), such Fe_3_O_4,_ to improve the separation performance of mixed matrix membranes (MMMs) has drawn a lot of interest to their integration into polymeric membranes. It is crucial to stress that physical sieving, which rejects macromolecules and particles based on pore size and distribution within the polymer matrix—remains the major retention mechanism of polymeric membranes. Additional features that enhance this sieving mechanism are provided by the embedded MNPs. In particular, because of their hydroxyl-rich surfaces, Fe_3_O_4_ nanoparticles offer surface adsorption sites that allow for improved removal of charged or polar contaminants through adsorption and electrostatic interactions. This improves the membrane's ability to reject colors, heavy metals, and organic pollutants without changing the membrane's basic sieving process. Therefore, rather than altering the main retention mechanism, the addition of nanoparticles improves membrane function. MNPs can occasionally take part in more complex oxidation processes like electrochemical oxidation or photocatalysis, however these reactions are conditional and only take place in response to external stimuli like light or applied potential or when combined with photocatalytic materials. These procedures do not take the place of the primary filtration and adsorption mechanisms; rather, they constitute an addition to them. Although MNPs enhance membrane performance, it is important to take into account their possible effects on the environment and biology. Reactive oxygen species (ROS), such as hydroxyl radicals (˙OH), hydrogen peroxide (H_2_O_2_), and superoxide anions (O_2_^−^), can be produced by Fe_3_O_4_ nanoparticles under oxidative circumstances.^157^Fig. 8 shows cell destruction caused by nanoparticles and clarifies the general mechanism of nanoparticle toxicity. These ROS can cause cytotoxic effects like genotoxicity, apoptosis, or other cellular stress responses. *In vitro* exposure of THP-1 cells to Fe_3_O_4_ nanoparticles at 25 µg mL^−1^ can result in cellular damage, according to toxicity studies, whereas *in vivo* tests in rats demonstrate apoptosis at 12 mg kg^−1^. These results emphasize how crucial it is to regulate the concentration of nanoparticles, guarantee stable integration within the polymer matrix, and stop leaching during membrane operation in order to reduce biological and environmental concerns. In conclusion, adding magnetic nanoparticles to polymeric membranes offers two advantages: better membrane selectivity through the current sieving mechanism and increased pollutant removal through adsorption. In order to ensure safe and efficient use in water treatment and other industrial processes, proper design and handling are also required to reduce possible toxicity.^158,159^

In another study, it was found that coated Fe_3_O_4_ nanoparticles showed excellent biosafety compared to uncoated iron oxide nanoparticles, as assessed by biochemical and neurobehavioral assays.^161^ On the other hand, magnetic IONPs are considered the least toxic among other nanomaterials, because of their short-term, temporary effects, their low sensitivity to oxidation, and their lack of contribution to permanent organ damage.^162–164^ Where only high doses of 500 mg Fe per kg can cause pathological changes in the spleen and liver.^165^ Actually, addressing these limitations is crucial to ensuring safety when using magnetic IONPs across various fields.

## 13 Challenges and prospects in the application of magnetic MMMs

In recent years, there has been an expansion in nanomaterials technology and their synthesis methods, especially magnetic IONPs.^121^ These nanomaterials play a crucial role and have applications in various fields; however, challenges related to filler aggregation persist and require further study to achieve uniform dispersion and meet the growing demands of different industrial applications. There are multiple opportunities for designing multifunctional MNP-based MMMs that integrate photocatalytic, antimicrobial, and regenerative capabilities for sustainable industrial wastewater treatment. However, the researchers' concern must be to introduce not only a good separation from the magnetic MMMs but also the possibility of achieving permanent performance for these membranes, with emphasis on cost control. Nevertheless, the focus on developing the magnetic IONPs and their applications in various fields of our life must open the door to use greener chemicals and even methods in its fabrication to conserve and secure the ecosystem, especially when these materials applied in MMMs and their contribution in separation processes of wastewater treatments to reach the standard environmental requirements and consequently, to reduce as possible the environmental pollution.

## 14 Conclusion

In conclusion, this review highlighted the adaptability of magnetic IONPs for diverse applications in catalysis, sensors, drug delivery, imaging, and environmental remediation, particularly in membrane technology. Membranes are superior barriers for controlling the transport of components between phases and play an important role in industrial and selective separation processes. Its synthetic techniques of phase inversion, LBL self-assembly, and electrospinning have been crucial in advancing membrane technology for different separation processes. The study highlights the unique features of magnetic IONPs, biodegradability, and compatibility, which are considered very promising to be used in membrane technology and especially mixed matrix membranes (MMMs). Also, the synthetic methods for magnetic IONPs and the significant contribution of (MMMs) were very important in reducing fouling and nanofiller agglomeration problems. In fact, the adsorption power of these materials made them number one for pollutant removal in wastewater treatment and membrane separation processes.

## Conflicts of interest

The authors declare no conflicts of interest.

## Data Availability

No primary research results, software or code have been included, and no new data were generated or analyzed as part of this review.

## References

[cit1] Zhao G., Gao H., Qu Z., Fan H., Meng H. (2023). Anhydrous interfacial polymerization of sub-1 Å sieving polyamide membrane. Nat. Commun..

[cit2] Ghadhban M. Y., Rashid K. T., Abdulrazak A. A., Ibrahim I. T., Alsalhy Q. F., Shakor Z. M., Hamawand I. (2024). Modification of Polylactide-poly (butylene adipate-co-terephthalate) (PLA/PBAT) Mixed-Matrix Membranes (MMMs) with Green Banana Peel Additives for Oil Wastewater Treatment. Water.

[cit3] Shabeeb K. M., Noori W. A., Abdulridha A. A., Majdi H. S., Al-Baiati M. N., Yahya A. A., Rashid K. T., Németh Z., Hernadi K., Alsalhy Q. F. (2023). Novel partially cross-linked nanoparticles graft co-polymer as pore former for polyethersulfone membranes for dyes removal. Heliyon.

[cit4] Alsalhy Q. F., Mohammed A. A., Ahmed S. H., Rashid K. T., AlSaadi M. A. (2018). Estimation of nanofiltration membrane transport parameters for cobalt ions removal from aqueous solutions. Desalination Water Treat..

[cit5] Sarran M. A., AbdulRazak A. A., Abid M. F., Jawad Al-Bayati A. D., Rashid K. T., Shehab M. A., Mohammed H. H., Alsarayefi S., Alhafadhi M., Alktranee M. (2024). Oily Wastewater Treatment by Using Fe_3_O_4_/Bentonite in Fixed-Bed Adsorption Column. ChemEngineering.

[cit6] Shen Z., Kuang Y., Zhou S., Zheng J., Ouyang G. (2023). Preparation of magnetic adsorbent and its adsorption removal of pollutants: An overview. TrAC, Trends Anal. Chem..

[cit7] Lu N., Sun Y., Li Y., Liu Z., Wang N., Meng T., Luo Y. (2025). A Review on the Application of Magnetic Nanomaterials for Environmental and Ecological Remediation. Toxics.

[cit8] Byakodi M., Shrikrishna N. S., Sharma R., Bhansali S., Mishra Y., Kaushik A., Gandhi S. (2022). Emerging 0D, 1D, 2D, and 3D nanostructures for efficient point-of-care biosensing. Biosens. Bioelectron.: X.

[cit9] Xu Y., Qiu Z., Chen J., Ma B., Zou W., Yang Z., Dai R. (2025). Orderly stacked 3D-nanohelices interlayer boosts performance of reverse osmosis membranes for effective water purification. Energy Environ. Sustainability.

[cit10] Haider A. J., Al-Anbari R. H., Mohammed H. A., Ahmed D. S. (2018). Synthesis of Multi-Walled Carbon Nanotubes Decorated with Zinc Oxide Nanoparticles for Removal of Pathogenic Bacterial. TrAC, Trends Anal. Chem..

[cit11] Karthik K., Dhanuskodi S., Gobinath C., Prabukumar S., Sivaramakrishnan S. (2018). Nanostructured CdO-NiO Composite for Multifunctional Applications. J. Phys. Chem. Solids.

[cit12] Huo T., Xue X., Sun Y., Huang S., Pan Y., Li F. (2026). Glomerulus Inspired Charged Nanofibrous Membrane for High-Efficient Micro/Nano-Emulsion Separation. Adv. Funct. Mater..

[cit13] Yousif N. A., Al-Jawad S. M. H., Taha A. A., Stamatis H. (2023). A Review of Structure, Properties, and Chemical Synthesis of Magnetite Nanoparticles. J. Appl. Sci. Nanotechnol..

[cit14] Dawud H. H., Ali E. H. (2018). Evaluation of purified urease activity from *Proteus mirabilis* using iron oxide nanoparticles and measurement of urea concentration in blood. TrAC, Trends Anal. Chem..

[cit15] Wei W., Zhaohui W., Taekyung Y., Jiang C., Kim W. (2015). Recent progress on magnetic iron oxide nanoparticles: synthesis, surface functional strategies and biomedical applications. Sci. Technol. Adv. Mater..

[cit16] Mimouni I., Bouziani A., Naciri Y., Boujnah M., El Belghiti M. A., El A. M. (2022). Effect of heat treatment on the photocatalytic activity of α-Fe_2_O_3_ nanoparticles: towards diclofenac elimination. Environ. Sci. Pollut. Res..

[cit17] Moaca E. A., Coricovac E. D., Soica C. M., Pinzaru I. A., Pacurariu C. S., Dehelean C. A. (2018). Preclinical aspects on magnetic iron oxide nanoparticles and their interventions as anticancer agents: enucleation, apoptosis and other mechanism. Iron Ores Iron Oxide Mater..

[cit18] Quach T. P. T., Doan L. (2023). Surface Modifications of Superparamagnetic Iron Oxide Nanoparticles with Polyvinyl Alcohol, Chitosan, and Graphene Oxide as Methylene Blue Adsorbents. Coatings.

[cit19] Amuanyena M. O. N., Kandawa-Schulz M., Kwaambwa H. M. (2019). Magnetic Iron Oxide Nanoparticles Modified with Moringa Seed Proteins for Recovery of Precious Metal Ions. J. Biomaterials Nanobiotechnol..

[cit20] Glasgow W., Fellows B., Qi B., Darroudi T., Kitchens C., Ye L., Crawford T., Mefford O. (2016). Continuous synthesis of iron oxide (Fe_3_O_4_) nanoparticles *via* thermal decomposition. Particuology.

[cit21] Li H., Lu Z., Cheng G., Rong K., Chen F., Chen R. (2015). HEPES-involved Hydrothermal Synthesis of Fe_3_O_4_ Nanoparticles and Their Biological Application. RSC Adv..

[cit22] Karimzadeh I., Aghazadeh M., Doroudi T., Ganjali M. R., Kolivand P. H. (2017). Superparamagnetic Iron Oxide (Fe_3_O_4_) Nanoparticles Coated with PEG/PEI for Biomedical Applications: A Facile and Scalable Preparation Route Based on the Cathodic Electrochemical Deposition Method. Adv. Phys. Chem..

[cit23] Lakshmanan R., Okoli C., Boutonnet M., Järås S. (2014). Microemulsion prepared magnetic nanoparticles for phosphate removal: Time efficient studies. J. Environ. Chem. Eng..

[cit24] Costo R., Bello V., Robic C., Port M., Marco J. F., Morales M. P., Veintemillas -Verdaguer S. (2012). Ultrasmall Iron Oxide Nanoparticles for Biomedical Applications: Improving the Colloidal and Magnetic Properties. Langmuir.

[cit25] Neto D. M. A., Freire R. M., Gallo J., Freire T. M., Queiroz D. C., Ricardo N. M. P. S., Vasconcelos I. F., Mele G., Luigi C., Mazzetto S. E., Bañobre-López M., Fechine P. B. A. (2017). Rapid Sonochemical Approach Produces Functionalized Fe_3_O_4_ Nanoparticles with Excellent Magnetic, Colloidal, and Relaxivity Properties for MRI Application. J. Phys. Chem. C.

[cit26] Alawiyah F., Muflikhah L. W. Z., Sulungbudi G. T., Mujamilah P. E. G. R. (2020). Synthesis and characterization of magnetite (Fe_3_O_4_) *via* radiolytic reduction method. J. Phys.: Conf. Ser.

[cit27] Singh V. P., Singh C. P., Kumar S., Member S., Pandey S. K., Member S., Puneth D. (2024). Microwave-Assisted Synthesis and Characterization of Iron Oxide Nanoparticles for Advanced Biomedical Sensing Applications. IEEE Open J. Nanotechnol..

[cit28] Estévez M., Cicuéndez M., Crespo J., Serrano-López J., Colilla M., Fernández-Acevedo C., Oroz-Mateo T., Rada-Leza A., González B., Izquierdo-Barba I., Vallet-Regí M. (2023). Large-scale production of superparamagnetic iron oxide nanoparticles by flame spray pyrolysis: *In vitro* biological evaluation for biomedical applications. J. Colloid Interface Sci..

[cit29] Kurapov Y. A., Vazhnichaya E. M., Litvin S. E., Romanenko S. M., Didikin G. G., Devyatkina T. A., Mokliak Y. V., Oranskaya E. I. (2019). Physical Synthesis of Iron Oxide Nanoparticles and Their Biological Activity in Vivo. SN Appl. Sci..

[cit30] Khalil M. I. (2015). Co-precipitation in aqueous solution synthesis of magnetite nanoparticles using iron (III) salts as precursors. Arab. J. Chem..

[cit31] Samrot A. V., Shobana N., Sruthi P. D., Sahithya C. S. (2018). Utilization of chitosan-coated superparamagnetic iron oxide nanoparticles for chromium removal. Arab. J. Chem..

[cit32] Yazdani F., Seddigh M. (2016). Magnetite nanoparticles synthesized by co-precipitation method: The effects of various iron anions on specifications. Mater. Chem. Phys..

[cit33] Nicola R., Costişor O., Ciopec M., Negrea A., Lazău R., Ianăşi C., Picioruş E.-M., Len A., Almásy L., Szerb E. I., Putz A.-M. (2020). Silica-Coated Magnetic Nanocomposites for Pb^2+^ Removal from Aqueous Solution. Appl. Sci..

[cit34] Antarnusa G., Jayanti P. D., Denny Y. R., Suherman A. (2022). Utilization of co-precipitation method on synthesis of Fe_3_O_4_/PEG with different concentrations of PEG for biosensor applications. Materialia.

[cit35] Baumgartner J., Dey A., Bomans P., Coadou C. L., Fratzl P., Sommerdijk N. A. J. M., Faivre D. (2013). Nucleation and growth of magnetite from solution. Nat. Mater..

[cit36] Arshad N., Khan M., Waseem M., Afzal M., Alarifi A., Saleem R. S. Z. (2023). Functionalization of surfactant templated magnetite by chitosan and PEGylated/Chitosan – *In vitro* studies on drug loading, release and anti-proliferative activity. J. Saudi Chem. Soc..

[cit37] Wu W., Wu Z., Yu T., Jiang C., Kim W. S. (2015). Recent progress on magnetic iron oxide nanoparticles: Synthesis, surface functional strategies and biomedical applications. Sci. Technol. Adv. Mater..

[cit38] Thamir A. D., Sukkar K. A., Ati A. A. (2017). Improve the Process of Enhancing Oil Recovery (EOR) by Applying Nanomagnetic Cobalt Ferrite Nanoparticles. TrAC, Trends Anal. Chem..

[cit39] Qiu L., Zhou S., Li Y., Rui W., Cui P., Zhang C., Yu Y., Wang C., Wang X., Wang J., Jiang P. (2021). Silica-Coated Fe_3_O_4_ Nanoparticles as a Bifunctional Agent for Magnetic Resonance Imaging and ZnII Fluorescent Sensing. Technol. Cancer Res. Treat..

[cit40] An G. S., Shin J. R., Hur J. U., Oh A. H., Kim B.-G., Jung Y.-G., Choiet S.-C. (2019). Fabrication of core–shell structured Fe_3_O_4_@Au nanoparticle *via* self-assembly method based on positively charged surface silylation/polymerization. J. Alloys Compd..

[cit41] Darmiyan H. R., Khosravi R., Shahryari T. (2022). Synthesis and characterization
of magnetic iron sulfide (Fe-S)/CuS nanocomposite as a novel recyclable catalyst in photocatalyst removal of tetracycline from aqueous solutions. Desalination Water Treat..

[cit42] Salman G. K., Bohan A. J., Jaed G. M. (2017). Use of Nano-Magnetic Material for Removal of Heavy Metals from Wastewater. TrAC, Trends Anal. Chem..

[cit43] Zakariya N. A., Jusof W. H. W., Majeed S. (2022). Green Approach for Iron Oxide Nanoparticles Synthesis: Application in Antimicrobial and Anticancer- an Updated Review. Karbala Int. J. Mod. Sci..

[cit44] Roy S. D., Das K. C., Dhar S. S. (2021). Conventional to green synthesis of magnetic iron oxide nanoparticles; its application as catalyst, photocatalyst and toxicity: A short review. Inorg. Chem. Commun..

[cit45] Al-Masri M., Amin Y., Al-Khateeb Y. (2023). Synthesis and Characterization of Green Magnetic Iron Oxide Nanoparticles for Ra Removal. Iran. J. Chem. Chem. Eng..

[cit46] Üstün E., Onbas S. C., Çelik S. K., Ayvaz M. Ç., ahin N. (2022). S¸ Green synthesis of iron oxide nanoparticles by using ficus carica leaf extract and its antioxidant activity. Biointerface Res. Appl. Chem..

[cit47] Iqbal J., Abbasi B. A., Ahmad R., Shahbaz A., Zahra S. A., Kanwal S., Munir A., Rabbani A., Mahmood T. (2020). Biogenic synthesis of green and cost effective iron nanoparticles and evaluation of their potential biomedical properties. J. Mol. Struct..

[cit48] Shejawal K. P., Randive D. S., Bhinge S. D., Bhutkar M. A., Wadkar G. H., Jadhav N. R. (2020). Green synthesis of silver and iron nanoparticles of isolated proanthocyanidin: its characterization, antioxidant, antimicrobial, and cytotoxic activities against COLO320DM and HT29. J. Genet. Eng. Biotechnol..

[cit49] Salim S., Hari N., Sudhi S., Nair A. J. (2025). Green synthesis, characterisation and bioactivity of iron oxide nanoparticles using Myristica fragrans leaf extract. The Microbe.

[cit50] Zúñiga-Miranda J., Guerra J., Mueller A., Mayorga-Ramos A., Carrera-Pacheco S. E., Barba-Ostria C., Heredia-Moya J., Guamán L. P. (2023). Iron Oxide Nanoparticles: Green Synthesis and Their Antimicrobial Activity. Nanomaterials.

[cit51] Brollo M. E. F., Orozco-Henao J. M., López-Ruiz R., Muraca D., Dias C. S. B., Pirota K. R., Knobel M. (2016). Magnetic hyperthermia in brick-like Ag@Fe_3_O_4_ core–shell nanoparticles. J. Magn. Magn. Mater..

[cit52] Tarkistani M. A. M., Komalla V., Kayser V. (2021). Recent advances in the use of iron–gold hybrid nanoparticles for biomedical applications. Nanomaterials.

[cit53] Wulandari I. O., Mardila V. T., Santjojo D., Sabarudin A. (2018). Preparation and Characterization of Chitosan-coated Fe_3_O_4_ Nanoparticles using Ex-Situ Co-Precipitation Method and Tripolyphosphate/Sulphate as Dual Crosslinkers. IOP Conf. Ser. Mater. Sci. Eng..

[cit54] An G. S., Shin J. R., Hur J. U., Oh A. H., Kim B.-G., Jung Y.-G., Choiet S.-C. (2019). Fabrication of core–shell structured Fe_3_O_4_@Au nanoparticle *via* self-assembly method based on positively charged surface silylation/polymerization. J. Alloys Compd..

[cit55] Mikhaylov V. I., Martakov I. S., Gerasimov E. Y., Sitnikov P. A. Study of heteroaggregation and properties of sol-gel AlOOH–Fe_3_O_4_ composites. 2020. Heliyon.

[cit56] Qiu L., Zhou S., Li Y., Rui W., Cui P., Zhang C., Yu Y., Wang C., Wang X., Wang J., Jiang P. (2021). Silica-Coated Fe_3_O_4_ Nanoparticles as a Bifunctional Agent for Magnetic Resonance Imaging and ZnII Fluorescent Sensing. Technol. Cancer Res. Treat..

[cit57] Wy W., Wu Z., Yu T., Jiang C., Kim W.-S. (2015). Recent progress on magnetic iron oxide nanoparticles: synthesis, surface functional strategies and biomedical applications. Sci. Technol. Adv. Mater..

[cit58] Arshad N., Khan M., Waseem M., Afzal M., Alarifi A., Saleem R. S. Z. (2023). Functionalization of surfactant templated magnetite by chitosan and PEGylated/Chitosan – *In vitro* studies on drug loading, release and anti-proliferative activity. J. Saudi Chem. Soc..

[cit59] Khan A. , Malik S., Bilal M., Ali N., Ni L., Gao X. and Hong K., 12 - Polymer-coated magnetic nanoparticles. Biopolymeric Nanomaterials Fundamentals and Applications A volume in Micro and Nano Technologies, Elsevier, 2021, pp. 275–292, 10.1016/B978-0-12-824364-0.00019-8

[cit60] Mylkie K., Nowak P., Rybczynski P., Ziegler-Borowska M. (2021). Polymer-Coated Magnetite Nanoparticles for Protein Immobilization. Materials.

[cit61] Kumar P., Tomar V., Kumar D., Joshi R. K., Nemiwal M. (2022). Magnetically active iron oxide nanoparticles for catalysis of organic transformations: A review. Tetrahedron.

[cit62] khalilifard M., Javadian S. (2021). Magnetic superhydrophobic polyurethane sponge loaded with Fe_3_O_4_@oleic acid@ graphene oxide as high performance adsorbent oil from water. Chem. Eng. J..

[cit63] Gu H., Fu S., Cai Z., Ai H. (2024). Polymer/iron oxide nanocomposites as magnetic resonance imaging contrast agents: Polymer modulation and probe property control. J. Polym. Sci..

[cit64] Peng L., Luo Y., Xiong H., Yao S., Zhu M., Song H. (2020). A Novel Amperometric Glucose Biosensor Based on Fe_3_O_4_-Chitosan-β-Cyclodextrin/MWCNTs Nanobiocomposite. Electroanalysis.

[cit65] Naznin A., Dhar P. K., Dutta S. K., Chakrabarty S., Karmakar U. K., Kundu P., Hossain M. S., Barai H. R., Haque M. R. (2023). Synthesis of Magnetic Iron Oxide-Incorporated Cellulose Composite Particles: An Investigation on Antioxidant Properties and Drug Delivery Applications. Pharmaceutics.

[cit66] Lu A. H., Salabas E. L., Schüth F. (2007). Magnetic nanoparticles: synthesis, protection, functionalization, and application. Angew Chem. Int. Ed. Engl..

[cit67] Kumar R. S., Arthanareeswaran G., Paul D., Ji H. K. (2015). Modification methods of polyethersulfone membranes for minimizing fouling–Review. Membr. Water Treat..

[cit68] Chaudhari D. S., Upadhyay R. P., Shinde G. Y., Gawande M. B., Filip J., Varma R. S., Zbořil R. (2024). A review on sustainable iron oxide nanoparticles: syntheses and applications in organic catalysis and environmental remediation. Green Chem..

[cit69] Roy S. D., Das K. C., Dhar S. S. (2021). Conventional to green synthesis of magnetic iron oxide nanoparticles; its application as catalyst, photocatalyst and toxicity: A short review. Inorg. Chem. Commun..

[cit70] Khan F. S. A., Mubarak N. M., Khalid M., Walvekar R., Abdullah E. C., Mazari S. A., Nizamuddin S., Karri R. R. (2020). Magnetic nanoadsorbents' potential route for heavy metals removal-a review. Environ. Sci. Pollut. Res..

[cit71] Rangelow I., Kaestner M., Ivanov T., Ahmad A., Lenk S., Lenk C., Guliyev E., Reum A., Hofmann M., Reuter C., Holz M. (2018). Atomic force microscope integrated with a scanning electron microscope for correlative nanofabrication and microscopy. J. Vac. Sci. Technol. B.

[cit72] Undavalli V. K. , Ling C. and Khandelwal B., Chapter 6 - Impact of alternative fuels and properties on elastomer compatibility, in Aviation Fuels, Academic Press. 2021, pp. 113–132, 10.1016/B978-0-12-818314-4.00001-7

[cit73] Ashizawa K. (2019). Nanosize Particle Analysis by Dynamic Light Scattering (DLS). Yakugaku Zasshi.

[cit74] Roca A. G., Gutiérrez L., Gavilán H., Brollo M. E. F., Veintemillas-Verdaguer S., del Puerto Morales M. (2019). Design Strategies for Shape-Controlled Magnetic Iron Oxide Nanoparticles. Adv. Drug Deliv. Rev..

[cit75] Glasgow W., Fellows B., Qi B., Darroudi T., Kitchens C., Ye L., Crawford T. M., Mefford O. T. (2016). Continuous synthesis of iron oxide (Fe_3_O_4_) nanoparticles *via* thermal decomposition. Particuology.

[cit76] Magalhães-Ghiotto G. A. V., Oliveira A. M. D., Natal J. P. S., Bergamasco R., Gomes R. G. (2021). Green nanoparticles in water treatment: a review of research trends, applications, environmental aspects and large-scale production. Environ. Nanotechnol. Monit. Manag..

[cit77] Sabri A. A., Albayati T. M., Abed D. B. (2018). Removal of Cobalt (Co(II)) from Aqueous Solution by Amino Functionalized SBA-15. TrAC, Trends Anal. Chem..

[cit78] Bassim S., Mageed A. K., AbdulRazak A. A., Majdi H. S. (2022). Green Synthesis of Fe_3_O_4_ Nanoparticles and Its Applications in Wastewater Treatment. Inorganics.

[cit79] Asrarian R., Jadidian R., Parham H., Haghtalab S. (2013). Removal of Aluminum from Water and Wastewater Using Magnetic Iron Oxide Nanoparticles. Adv. Mater. Res..

[cit80] Rashid U. S., Saini-Eidukat B., Bezbaruah A. N. (2020). Modeling arsenic removal by nanoscale zero-valent iron. Environ. Monit. Assess..

[cit81] Bilgiç A., Karapınar H. S. (2022). APTMS-BCAD modified magnetic iron oxide for magnetic solid-phase extraction of Cu(II) from aqueous solutions. Heliyon.

[cit82] Cusioli L. F., Quesada H. B., de Brito Portela Castro A. L., Gomes R. G., Bergamasco R. (2020). Development of a new low-cost adsorbent functionalized with iron nanoparticles for removal of metformin from contaminated water. Chemosphere.

[cit83] Nguyen M. D., Tran H. V., Xu S., Lee T. R. (2021). Fe_3_O_4_ Nanoparticles: Structures, Synthesis, Magnetic Properties, Surface Functionalization, and Emerging Applications. Appl. Sci..

[cit84] Sinha S., Sharma R., Ansari M. R., Singh R., Pathak S., Jahan N., Rao Peta K. (2025). Multifunctional oleic acid functionalized iron oxide nanoparticles for antibacterial and dye degradation applications with magnetic recycling. Mater. Adv..

[cit85] Zinadini S., Zinatizadeh A. A., Rahimi M., Vatanpour V., Zangeneh H., Beygzadeh M. (2014). Novel high flux antifouling nanofiltration membranes for dye removal containing carboxymethyl chitosan coated Fe_3_O_4_ nanoparticles. Desalination.

[cit86] Khelali A., Benmahdi F., Khettaf S., Farooq S., Hacıosmanoğlu G. G. (2025). “Synthesis and characterization of a high-performance magnetic nanocomposite adsorbent from pomegranate pomace waste for tannery wastewater treatment. Surf. Interfaces.

[cit87] Al-Khateeb L. A., El-Maghrabey M., Alshaikhi D., Kashmery H. A., Alsuraihy R. H., El-Shaheny R. (2025). Magnetic Fe_3_O_4_NPs@nanoclay composite for efficient adsorptive removal of NSAIDs from environmental water with kinetic study of the adsorption process. Microchem. J..

[cit88] Talebi J., Zirar F.-E., El-Asri A., El Hayaoui W., Wail El Mouhri W., Nadif I., Tajat N., Bouddouch A., Tamimi M., Qourzal S., Assabbane A., Bakas I. (2025). Synthesis and investigation of a polypyrrole–magnetic oxide–graphene (FOPYGO) nanocomposite for efficient dye removal from wastewater: experimental and theoretical (DFT & Monte Carlo) studies. Appl. Surf. Sci..

[cit89] Su T., Zhang X., Wang Z., Guo Y., Wei X., Xu B., Xia H., Yang W., Xu H. (2024). Cellulose nanocrystal-based polymer hydrogel embedded with iron oxide nanorods for efficient arsenic removal. Carbohydr. Polym..

[cit90] Othman I., Banat F., Hasan S. W., Aubry C., Suresh S., Sillanpää M., Abu Haija M. (2023). Facile Preparation of Magnetic CuFe_2_O_4_ on Sepiolite/GO Nanocomposites for Efficient Removal of Pb(II) and Cd(II) from Aqueous Solution. ACS Omega.

[cit91] Metta S. R., Sahu U. K., Sahu M. K., Mohanty H. S., Kar P. (2025). Application of Box-Behnken Design Model for Statistical Optimization of As(III) Removal Using Iron Manganese Bimetal Oxide Composite. ChemistrySelect.

[cit92] Chan K., Zinchenko A. (2022). Conversion of waste bottle PET to magnetic microparticles adsorbent for dye-simulated wastewater treatment. J. Environ. Chem. Eng..

[cit93] Ahmed M. A., Ahmed M. A., Mohamed A. A. (2023). Removal of 4-nitrophenol and indigo carmine dye from wastewaters by magnetic copper ferrite nanoparticles: Kinetic, thermodynamic and mechanistic insights. J. Saudi Chem. Soc..

[cit94] Chkirida S., El Mernissi N., Zari N., Qaiss A., Bouhfid R. (2024). In-situ magnetic alginate coated chitosan core@shell beads with excellent performance in simulated and real wastewater treatment: Behavior, mechanisms, and new perspectives. Int. J. Biol. Macromol..

[cit95] Hossain M. S., Kabir M. H., Ali S. M. A., Haque M. A., Yasmin S. (2024). Ultrafast and simultaneous removal of four tetracyclines from aqueous solutions using waste material-derived graphene oxide-supported cobalt–iron magnetic nanocomposites. Electronic supplementary information (ESI)available. RSC Adv..

[cit96] Arabkhani P., Asfaram A. (2025). A novel biowaste-derived magnetic adsorbent for efficient removal of cadmium, cobalt and strontium ions from industrial wastewater. Inorg. Chem. Commun..

[cit97] Mpelane S., Mketo N., Bingwa N., Nomngongo P. N. (2022). Synthesis of mesoporous iron oxide nanoparticles for adsorptive removal of levofloxacin from aqueous solutions: Kinetics, isotherms, thermodynamics and mechanism. Alex. Eng. J..

[cit98] Wang Y., Zhang Y., Liang L., Tu F., Li Z., Tang X., Dai L., Li L. (2024). Research Progress on Membrane Separation Technology for Oily Wastewater Treatment. Toxics.

[cit99] Nthunya L. N., Chong K. C., Lai S. O., Lau W. J., Lopez-Maldonado E. A., Camacho L. M., Shirazi M. M. A., Ali A., Mamba B. B., Osial M., Pietrzyk-Thel P., Pregowska A., Mahlangu O. T. (2024). Progress in membrane distillation processes for dye wastewater treatment: A review. Chemosphere.

[cit100] Keskin B., Zeytuncu-Gökŏ glu B., Koyuncu I. (2021). Polymer inclusion membrane applications for transport of metal ions: A critical review. Chemosphere.

[cit101] Baig N., Kammakakam I., Falath W., Kammakakam I. (2021). Nanomaterials: A Review of Synthesis Methods, Properties, Recent Progress, and Challenges. Mater. Adv..

[cit102] Dmitrieva E. S., Anokhina T. S., Novitsky E. G., Volkov V. V., Borisov I. L., Volkov A. V. (2022). Polymeric Membranes for Oil-Water Separation: A Review. Polymers.

[cit103] Chen M., Heijman S. G. J., Luiten-Olieman M. W. J., Rietveld L. C. (2022). Oil-in-water emulsion separation: Fouling of alumina membranes with and without a silicon carbide deposition in constant flux filtration mode. Water Res..

[cit104] Arumugham T., Kaleekkal N. J., Gopal S., Nambikkattu J., Rambabu K., Aboulella A. M., RanilWickramasinghe S., Banat F. (2021). Recent developments in porous ceramic membranes for wastewater treatment and desalination: A review. J. Environ. Manag..

[cit105] Kesting R. E. and Fritzsche A., Polymeric Gas Separation Membranes, Wiley-Interscience, 1993, 10.1002/pi.1995.210360116

[cit106] Asif M. B., Zhang Z. (2021). Ceramic membrane technology for water and wastewater treatment: A critical review of performance, full-scale applications, membrane fouling and prospects. Chem. Eng. J..

[cit107] Petra C. and Katalin B.-B., Application of Ionic Liquids in Membrane Separation Processes, in. Ionic Liquids: Applications and Perspectives, ed. A. Kokorin, ISBN: 978-953-307-248-7, InTech, 2011, available from: https://www.intechopen.com/books/ionic-liquids-applications-and-perspectives/application-ofionic-liquids-in-membrane-separation-processes

[cit108] El-Sheekh M. M., El-Kassas H. Y., Shams El-Din N. G., Eissa D. I., El-Sherbiny B. A. (2021). Green synthesis, characterization applications of iron oxide nanoparticles for antialgal and wastewater bioremediation using three brown algae. Int. J. Phytomed..

[cit109] Sonawane A. V., Murthy Z. V. P. (2022). Synthesis, characterization, and application of ZIF-8/Ag_3_PO_4_, MoS_2_/Ag_3_PO_4_, and h-BN/Ag_3_PO_4_ based photocatalytic nanocomposite polyvinylidene fluoride mixed matrix membranes for effective removal of drimaren orange P2R. J. Membr. Sci..

[cit110] Gandomkar E., Fazlali A. (2023). Small but Mighty Incorporating Fe_3_O_4_ Nanoparticles into PES Membranes for Enhanced Water Treatment Efficiency. Iran. J. Chem. Chem. Eng..

[cit111] Cheng Y., Joarder B., Datta S. J., Alsadun N., Poloneeva D., Fan D., Khairova R., Bavykina A., Jia J., Shekhah O., Shkurenko A., Maurin G., Gascon J., Eddaoudi M. (2023). Mixed Matrix Membranes with Surface Functionalized Metal-Organic Framework Sieves for Efficient Propylene/Propane Separation. Adv. Mater..

[cit112] Diwan T., Abudi Z. N., Al-Furaiji M. H., Nijmeijer A. (2023). A Competitive Study
Using Electrospinning and Phase Inversion to Prepare Polymeric Membranes for Oil Removal. Membranes.

[cit113] Hu C.-C., Cheng P.-H., Chou S.-C., Lai C.-L., Huang S.-H., Tsai H.-A., Hung W.-S., Lee K.-R. (2020). Separation behavior of amorphous amino-modified silica nanopar-ticle/polyimide mixed matrix membranes for gas separation. J. Membr. Sci..

[cit114] Rahman M. T., Hoque M. D. A., Rahman G. T., Azmi M. M., Gafur M. A., Khan R. A., Hossain M. K. (2019). Fe_2_O_3_ nanoparticles dispersed unsaturated polyester resin based nanocomposites: effect of gamma radiation on mechanical properties. Radiat. Eff. Defect Solid.

[cit115] Asadul Hoque Md., Ahmed M. R., Rahman G. T., Rahman M. T., Islam M. A., Khan M. A., Khalid Hossain M. (2018). Fabrication and comparative study of magnetic Fe and α-Fe_2_O_3_ nanoparticles dispersed hybrid polymer (PVA+Chitosan) novel nanocomposite film. Results Phys..

[cit116] Hardian R., Jia J., Diaz-Marquez A., Naskar S., Fan D., Shekhah O., Maurin G., Eddaoudi M., Szekely G. (2024). Design of Mixed-Matrix MOF Membranes with Asymmetric Filler Density and Intrinsic MOF/Polymer Compatibility for Enhanced Molecular Sieving. Adv. Mater..

[cit117] Zhang M., Jiang S., Guo X., Tang X., Bai L., Wang J., Zhang H., Xu D., Wu R., Liu L., Liang H. (2023). Fabrication of high permeability and antifouling composite membrane loaded with Fe_3_O_4_ nanoparticles *via* a magnetic field induced phase separation. J. Water Proc. Eng..

[cit118] Mao G., Kan Q., Yue Z., Ju S., Zhou H., Jin W. (2025). In situ polymerization of UiO-66-NH_2_ with polyamide to fabricate mixed-matrix membranes with enhanced separation performance for methanol and dimethyl carbonate. Sep. Purif. Technol..

[cit119] Kelarakis A. (2024). In Situ Generation of Nanoparticles on and within Polymeric Materials. Polymers.

[cit120] Nikpour N., Montazer A. H., Khayatian A. (2022). Magnetic field-induced improvement in O_2_/N_2_ gas separation applications of simultaneously co-casted superparamagnetic mixed matrix membranes. J. Ind. Eng. Chem..

[cit121] Wang Y., Gu X., Quan J., Xing G., Yang L., Zhao C., Wu P., Zhao F., Hu B., Hu Y. (2021). Application of magnetic fields to wastewater treatment and its mechanisms: A review. Sci. Total Environ..

[cit122] Hafiz M. A., Hassanein A., Talhami M., AL-Ejji M., Hassan M. K., Hawari A. H. (2022). Magnetic nanoparticles draw solution for forward osmosis: Current status and future challenges in wastewater treatment. J. Environ. Chem. Eng..

[cit123] Li R., Chen J. P., Freger V. (2023). A new fabrication approach for mixed matrix membrane fabricated with interstitially sealed MOF nanoparticles. J. Membr. Sci..

[cit124] Almomani F., Bhosale R., Khraisheh M., kumar A., Almomani T. (2022). Heavy metal ions removal from industrial wastewater using magnetic nanoparticles (MNP). Appl. Surf. Sci..

[cit125] Suryanto H., Syukri D., Kurniawan F., Yanuhar U., Binoj J. S., Efendi S., Nusantara F., Maulana J., Caesar N. R., Komarudin K. (2024). Enhanced Dye Adsorption and Bacterial Removal of Magnetic Nanoparticle-Functionalized Bacterial Cellulose Acetate Membranes. J. Renew. Mater..

[cit126] Ismail N. H., Salleh W. N. W., Rosman N., Awang N. A., Hasbullah H., Aziz F., Yusof N. (2019). PVDF/Fe_2_O_3_ mixed matrix membrane for oily wastewater treatment. Malays. J. Fundam. Appl. Sci..

[cit127] Yang X., Wen Y., Li Y., Yan L., Tang C. Y., Ma J., Darling S. B., Shao L. (2023). Engineering *In Situ* Catalytic Cleaning Membrane *Via* Prebiotic-Chemistry-Inspired Mineralization. Adv. Mater..

[cit128] Yang X., Sun P., Wen Y., Mane A. U., Elam J. W., Ma J., Liu S., Darling S. B., Shao L. (2024). Protein-activated atomic layer deposition for robust crude-oil-repellent hierarchical nano-armored membranes. Sci. Bull..

[cit129] Yang X., Li Y., Wu D., Yan L., Guan J., Wen Y., Bai Y., Mamba B. B., Darling S. B., Shao L. (2024). Chelation-directed interface engineering of in-place self-cleaning membranes. Proc. Natl. Acad. Sci. U. S. A..

[cit130] Wang H., Wang X., Yu Y., Mamba B. B., Jiang X., Yang X., Shao L. (2026). Engineering self-rebound catalytic membranes for efficient high-viscosity oily wastewater purification and emerging contaminants removal. Water Res..

[cit131] Li Y., Sun P., Guo J., Lei C., Nxumalo E. N., Mamba B. B., Yang X., Jiang X., Shao L. (2025). Ultrarobust biomimetic mineralized membranes *via* heterophase interface engineering. Matter.

[cit132] Mirzaei M., Mohammadi T., Kasiri N., Tofighy M. A. (2021). Fabrication of magnetic field induced mixed matrix membranes containing GO/Fe_3_O_4_ nanohybrids with enhanced antifouling properties for wastewater treatment applications. J. Environ. Chem. Eng..

[cit133] Hosseini S. M., Afshari M., Fazlali A. R., Farahani S., Bandehali S., Van der Bruggen B., Bagheripour E. (2019). Mixed matrix PES-based nanofiltration membrane decorated by (Fe_3_O_4_-polyvinylpyrrolidone) composite nanoparticles with intensified antifouling and separation characteristics. Chem. Eng. Res. Des..

[cit134] Nawi N. S. M., Jye L. W., Yusof N., Said N., Ismail A. (2021). Surface Modification of PES Hollow Fiber Membranes using Iron Oxide Particles for Water Treatment: Does Particle Size Really Matter?. Malays. J. Fundam. Appl. Sci..

[cit135] Evangeline C., Pragasam V., Rambabu K., Velu S., Monash P., Arthanareeswaran G., Banat F. (2019). Iron oxide modified polyethersulfone/cellulose acetate blend membrane for enhanced defluoridation application. Desalination Water Treat..

[cit136] Jawad S. K., Rashid K. T., Toma M. A., Abdul Razak A. A., Shehab M. A., Ghadhban M. Y., Al-lami M., Alhafadhi M., Mohammed H. H., Hmood A. A., Al-Ogaili M. F. A., Alsarayefi S. (2025). Optimal operating conditions of PES/BiFeO_3_ mixed matrix membrane for treating dye-contaminated wastewater. Results Eng..

[cit137] Abomostafa H. M., Isawi H., Dalia E., Abulyazied D. E., Abouhaswa A. S. (2023). Advanced photocatalytic degradation of organic pollutants using magnetic nanostructured PVA membrane under solar irradiation. Surf. Interfaces.

[cit138] Bhavani D. H., Manjunatha Kumara K. S., Padaki M., Nagaraju D. H. (2025). Tuning the membrane surface charge: Zwitterionic functionalized iron oxide nanoparticles for molecular separation and their superior antifouling property. J. Environ. Chem. Eng..

[cit139] Lakhotia S. R., Mukhopadhyay M., Kumari P. (2019). Iron oxide (FeO) nanoparticles embedded thin-film nanocomposite nanofiltration (NF) membrane for water treatment. Sep. Purif. Technol..

[cit140] Bedar A., Yadav D., Kirti S., Das R. K., Saxena S., Shukla S. (2024). Nanomagnets doped antifouling membrane for fine emulsion separation. Polymer.

[cit141] Ghaemi N., Madaeni S. S., Daraei P., Rajabi H., Zinadini S., Alizadeh A., Heydari R., Beygzadeh M., Ghouzivand S. (2015). Polyethersulfone membrane enhanced with iron oxide nanoparticles for copper removal from water: Application of new functionalized Fe_3_O_4_ nanoparticles. Chem. Eng. J..

[cit142] Al-Husaini I. S., Yusoff A.-R. M., Lau W.-J., Ismail A. F., Al-Abri M. Z., Wirzal M. D. H. (2019). Iron oxide nanoparticles incorporated polyethersulfone electrospun nanofibrous membranes for effective oil removal. Chem. Eng. Res. Des..

[cit143] Abdel-Karim A., Ismail S. H., Bayoumy A. M., Ibrahim M., Mohamed G. G. (2021). Antifouling PES/Cu@Fe_3_O_4_ mixed matrix membranes: Quantitative structure–activity relationship (QSAR) modeling and wastewater treatment potentiality. Chem. Eng. J..

[cit144] Vafaee B. M., Omidvar M., Zhiani R., Hosseiny M., Nouri S. M. M. (2024). Application of Novel Immobilized Fe_3_O_4_ Nanoparticles in Mixed Matrix Polyethersulfone Membrane for Oily Wastewater Treatment. Iran. J. Chem. Chem. Eng..

[cit145] Isawi H. (2024). Using zero nano-valent iron/thin film composite (ZNVI/TFC) membrane for brackish water desalination and purification. Surf. Interfaces.

[cit146] Mirzaei M., Mohammadi T., Kasiri N., Tofighy M. A. (2021). Fabrication of magnetic field induced mixed matrix membranes containing GO/Fe_3_O_4_ nanohybrids with enhanced antifouling properties for wastewater treatment applications. J. Environ. Chem. Eng..

[cit147] Ibrahim H. A., Rashid K. T., AbdulRazak A. A., Shehab M. A., Salih M. A., Al-lami M., Al-Mayyahi M. A. T., Al-Ashoor S. Z., Shakir S. K., Mohammed H. H., Mahmood A. (2025). A novel poly(ether-Sulfone) mixed matrix membranes infused with TiO_2_-BiFeO_3_ nanomaterials for the removal of toxic Congo red dye from textile wastewater. Chem. Eng. J. Adv..

[cit148] Mokubung K. E., Madzivha N. N., Lau W. J., Nxumalo E. N. (2025). Polyethersulfone/biochar-Fe_3_O_4_/GO mixed matrix membranes with enhanced antifouling properties for heavy metals removal from acid mine drainage. Inorg. Chem. Commun..

[cit149] Kusworo T. D., Kumoro A. C., Buchori L., Faturahman F., Alfaridzi J., Othman M. H. D., Kurniawan T. A., Utomo D. P. (2025). Enhancing sulfonamide antibiotic wastewater treatment: A novel approach integrating ozone-assisted kaolin adsorption and PSf/TiO_2_-NiFe_2_O_4_@SiO_2_ photocatalytic hybrid membrane. J. Water Proc. Eng..

[cit150] Mokubung K. E., Gumbi N. N., Lau W. J., Nxumalo E. N. (2024). Pine cone derived polyethersulfone/biochar-Fe_3_O_4_ mixed matrix membranes for removal of arsenic from acid mine
drainage. Chem. Eng. Res. Des..

[cit151] Suter E., Rutto H. L., Mkhize I. G. (2025). Biodegradable waste-derived cellulose/nylon-6-coated iron-oxide nanocomposite encapsulated with chitosan for enhanced wastewater treatment. Clean. Chem. Eng..

[cit152] Balogun H. A., Lively R. P. (2026). Independent experimental measurements of diffusion, sorption, and permeability support the solution-diffusion model of membrane transport. J. Membr. Sci..

[cit153] Ali I., Peng C., Naz I., Khan Z. M., Sultan M., Islam T., Abbasi I. A. (2017). Phytogenic magnetic nanoparticles for wastewater treatment: a review. RSC Adv..

[cit154] Ajith M. P., Aswathi M., Priyadarshini E., Rajamani P. (2021). Recent innovations of nanotechnology in water treatment: a comprehensive review. Bioresour. Technol..

[cit155] Deivayanai V. C., Thamarai P., Karishma S., Saravanan A., Vickram A. S., Yaashikaa P. R., Sonali S. (2025). A comprehensive review on impregnated magnetic nanoparticle in advanced wastewater treatment: An in-depth technical review and future directions. Sustainable Chem. Environ..

[cit156] Viteri A., Laugé J., Lutz L., Ginebra M.-P., García-Torres J. (2025). In situ synthesis of Fe_3_O_4_ nanocatalyst in chitosan-agarose hydrogel membranes for the sustainable and efficient degradation of organic compounds. Int. J. Biol. Macromol..

[cit157] AshaRani P. V., Sethu S., Lim H. K., Balaji G., Valiyaveettil S., Hande M. P. (2012). Differential regulation of intracellular factors mediating cell cycle, DNA repair andinflammation following exposure to silver nanoparticles in human cells. Genome Integr..

[cit158] Yu Z., Li Q., Wang J., Yu Y., Wang Y., Zhou Q., Li P. (2020). Reactive oxygen species related nanoparticle toxicity in the biomedical field. Nanoscale Res. Lett..

[cit159] Ran Q., Xiang Y., Liu Y., Xiang L., Li F., Deng X., Xiao Y., Chen L., Chen L., Li Z. (2015). Eryptosis indices as a novel predictive parameter for biocompatibility of Fe_3_O_4_ magnetic nanoparticles on erythrocytes. Sci. Rep..

[cit160] Awashra M., Młynarz P. (2023). The toxicity of nanoparticles and their interaction with cells: an *in vitro* metabolomic perspective. Nanoscale Adv..

[cit161] Malhotra N., Chen J.-R., Sarasamma S., Audira G., Siregar P., Liang S.-T., Lai Y.-H., Lin G.-M., Ger T.-R., Hsiao C.-D. (2019). Ecotoxicity assessment of fe3o4 magnetic nanoparticle exposure in adult zebrafish at an environmental pertinent concentration by behavioral and biochemical testing. Nanomaterials.

[cit162] Valdiglesias V., Fernandez-Bertolez N., Kiliç G., Costa C., Costa S., Fraga S., Bessa M. J., Pasaro E., Teixeira J. P., La_on B. (2016). Are iron oxide nanoparticles safe? Current knowledge and future perspectives. J. Trace Elem. Med. Biol..

[cit163] Aboushoushah S., Alshammari W., Darwesh R., Elbaily N. (2021). Toxicity and biodistribution assessment of curcumin-coated iron oxide nanoparticles: multidose administration. Life Sci..

[cit164] Sun Z., Worden M., Thliveris J. A., Hombach-Klonisch S., Klonisch T., van Lierop J., Hegmann T., Miller D. W. (2016). Biodistribution of negatively charged iron oxide nanoparticles (IONPs) in mice and enhanced brain delivery using lysophosphatidic acid (LPA). Nanomed.: Nanotechnol. Biol. Med..

[cit165] Katsnelson B. A., Degtyareva T. D., Minigalieva I. I., Privalova L. I., Kuzmin S. V., Yeremenko O. S., Kireyeva E. P., Sutunkova M. P., Valamina I. I., Khodos M. Y., Kozitsina A. N., Shur V. Y., Vazhenin V. A., Potapov A. P., Morozova M. V. (2011). Subchronic systemic toxicity and bioaccumulation of Fe_3_O_4_ nano- and microparticles following repeated intraperitoneal administration to rats. Int. J. Toxicol..

